# Intestinal cell diversity and treatment responses in a parasitic nematode at single cell resolution

**DOI:** 10.1186/s12864-024-10203-7

**Published:** 2024-04-04

**Authors:** Rahul Tyagi, Bruce A Rosa, Amanda Swain, Maxim N Artyomov, Douglas P Jasmer, Makedonka Mitreva

**Affiliations:** 1 Division of Infectious Diseases, Department of Internal Medicine, Washington University School of Medicine, 63110 St. Louis, MO USA; 2 Department of Pathology and Immunology, Washington University School of Medicine, 63110 Saint Louis, MO USA; 3 Department of Veterinary Microbiology and Pathology, Washington State University, 99164 Pullman, WA USA; 4 Department of Genetics, Washington University School of Medicine, 63110 St. Louis, MO USA; 5 McDonnell Genome Institute, Washington University School of Medicine, 63110 St Louis, MO USA

**Keywords:** Single-cell transcriptomics, Roundworm, Intestine, Parasitology

## Abstract

**Background:**

Parasitic nematodes, significant pathogens for humans, animals, and plants, depend on diverse organ systems for intra-host survival. Understanding the cellular diversity and molecular variations underlying these functions holds promise for developing novel therapeutics, with specific emphasis on the neuromuscular system’s functional diversity. The nematode intestine, crucial for anthelmintic therapies, exhibits diverse cellular phenotypes, and unraveling this diversity at the single-cell level is essential for advancing knowledge in anthelmintic research across various organ systems.

**Results:**

Here, using novel single-cell transcriptomics datasets, we delineate cellular diversity within the intestine of adult female *Ascaris suum*, a parasitic nematode species that infects animals and people. Gene transcripts expressed in individual nuclei of untreated intestinal cells resolved three phenotypic clusters, while lower stringency resolved additional subclusters and more potential diversity. Clusters 1 and 3 phenotypes displayed variable congruence with scRNA phenotypes of *C. elegans* intestinal cells, whereas the *A. suum* cluster 2 phenotype was markedly unique. Distinct functional pathway enrichment characterized each *A. suum* intestinal cell cluster. Cluster 2 was distinctly enriched for Clade III-associated genes, suggesting it evolved within clade III nematodes. Clusters also demonstrated differential transcriptional responsiveness to nematode intestinal toxic treatments, with Cluster 2 displaying the least responses to short-term intra-pseudocoelomic nematode intestinal toxin treatments.

**Conclusions:**

This investigation presents advances in knowledge related to biological differences among major cell populations of adult *A. suum* intestinal cells. For the first time, diverse nematode intestinal cell populations were characterized, and associated biological markers of these cells were identified to support tracking of constituent cells under experimental conditions. These advances will promote better understanding of this and other parasitic nematodes of global importance, and will help to guide future anthelmintic treatments.

**Supplementary Information:**

The online version contains supplementary material available at 10.1186/s12864-024-10203-7.

## Introduction

Parasitic nematodes are pathogens of high significance to the health of humans, animals and plants. These parasites rely on multiple organ systems to survive within the hosts that they infect. Resolution of the cellular diversity and associated molecular differences that underlie functions required for intra-host survival of parasitic nematodes offers insight that may have practical applications toward new therapeutics for treatment and control of these pathogens. The various organ systems that comprise individual parasitic nematodes account for substantial cellular diversity of these pathogens, while the diversity of cellular populations that comprise each organ system remains far less clear. The neuromuscular system of nematodes exemplifies the relevance in defining the functional diversity of cells comprising nematode organ systems. Subsets of neurons and the muscle groups they control have been mapped for some nematodes with respect to sensitivity and acquired resistance to various anthelmintics [1]. Cellular resolution of this kind has received far less attention in other organ systems of parasitic nematodes, although evidence supports that such clarification will have practical application.

The nematode intestine is another organ with documented application to anthelmintic therapies and it displays diversity in cellular phenotypes relevant to such therapies. Catastrophic destruction of intestinal cells can be induced by anthelmintics in parasitic nematodes [2]. Concurrently, cells situated in the anterior-most region of the *Haemonchus contortus* intestine showed hypersensitivity to the benzimidazole anthelmintic, fenbendazole [3]. Transcriptomic differences characterize three contiguous regions that comprise the *Ascaris suum* intestine [4], and gene expression differences characterize individual intestinal cells of *C. elegans* [5]. Thus, facts gathered from disparate parasitic and non-parasitic nematodes indicate a diversity of phenotypes among cells that comprise the nematode intestine, the details of which have likely relevance to anthelmintic therapies. Further, it is expected that each organ system in parasitic nematodes has intrinsic characteristics ultimately exploitable in anthelmintic research, and as with the intestinal and neuromuscular tissues, diversity of cellular phenotypes comprising each organ system may translate into greater or lesser sensitivity to given anthelmintics. Consequently, knowledge at single cell level, molecular explanations for anthelmintic sensitivities, and methods to extract this information are all goals worth working toward.

With these goals in mind, we have focused on establishing a model for the nematode intestine that supports anthelmintic research in a pan-Nematoda context. Progress has involved development and integration of multi-omics databases for the nematode intestine, and development of multiple computational tools and research methods that capitalize on these databases [2]. We identified multiple toxins for intestinal cells with broad phylogenetic application by integrating these databases with a systems biology approach and publicly accessible drug databases to elucidate specific intestinal targets and small molecule inhibitors [6]. Bulk transcriptomic analyses documented gene responses induced by these toxin treatments. Coupled with new methods, pathologic effects induced by selected toxins were detailed for the intestine, and results were extended to the cells of multiple organ systems in whole intact worm assays [7]. Notably, the broad cellular visualization afforded by these methods provided rapid determination of toxin-induced death of many cells across many organs, inclusive of neuronal degeneration, and in multiple distantly related species [7, 8].

Although variability in gene expression and anthelmintic-sensitivity phenotype has been documented along the length of the nematode intestine [4], detailed assessment of intestinal cell phenotypic diversity has not been accomplished for any parasitic nematode species. Given that phenotypic diversity can determine sensitivity to toxins, knowledge of cellular diversity within organs targeted in anthelmintic research is of basic interest. For instance, 50% efficacy of a treatment across all cells in a target organ might reflect 100% efficacy of the true “sensitive” subset of cells. Ability to catalogue and sort genes expressed by sensitive versus insensitive cells on a single cell basis may advance mechanistic explanations for cell toxicity and facilitate anthelmintic development in general. Such cellular resolution has not been possible in the past, but recent advances in single-cell sequencing analysis [9, 10] means that exploration of many issues of this kind are now within our grasp. Specifically, single cell RNA sequencing (scRNAseq) has been instrumental in gaining new insights into disease biology, including cancer [11], Alzheimer’s disease [12, 13], cardiovascular disease [14] and infectious diseases [15–17]. Further applications of scRNAseq have extended to delineating tissue heterogeneity and establishing cellular atlases in human and model organisms [18–25], including whole adult worm or embryos of *C. elegans* [5, 26, 27] and other helminths [28–33]. However, the only nematode with reported tissue-specific single-cell resolution analysis is the free-living nematode *C. elegans* [34].

Here we initiated research that begins to delineate cellular diversity of the intestine of *A. suum*, a parasitic nematode species that infects animals and people [35] and has been key to many aspects of nematode intestinal cell research [2]. In contrast to many species of more diminutive size, adult *A. suum* can reach 35 cm, which supports dissection of its intestine and other organs using macro methods. One consequence of such large size is the high number of cells comprising the *A. suum* intestine (of the order of millions, as enumerated in this paper), compared to just 20 cells comprising the *C. elegans* intestine. We developed methods to produce single-nuclei preparations from adult female *A. suum* intestinal cells, focusing on a region of the intestinal segment free from attachments from other tissues. Transcripts expressed in those nuclei were analyzed by scRNAseq for both untreated and toxin-treated adult *A. suum*, identifying and characterizing three major cell clusters and seven smaller subclusters. Our findings offer technical advancements applicable to other tissues and provide crucial insights into parasitic nematodes, enhancing our understanding of these globally significant parasites.

## Methods

### A. *suum* adult parasite sample preparation

Adult *A. suum* were collected from the intestines of swine that were processed for slaughter at the University of Idaho Meat Science Laboratory (Moscow, Idaho) and transported to the laboratory at Washington State University in warm phosphate buffered saline (PBS, pH 7.4, initially 37^o^C). The nematodes were then rinsed 3 times in PBS prior to immediate dissection or experimental treatment and dissection. To assess natural phenotypic diversity of cells that comprise the intestine, intestinal tissue was obtained from three untreated adult *A. suum* (sample names F1-UT, F2-UT and F3-UT in Supplementary Table S1) utilizing methods described previously [36]. Briefly, heads of worms are removed with a scalpel below the esophagus and tails removed just anterior to the anus. A 25-gauge needle-cannula is inserted into the anterior opening of the intestinal lumen and secured with superglue. Five mL PBS (37 ^o^C) was then gently perfused by syringe through the intestine to remove content from the intestinal lumen. Following perfusion, worms were further dissected to obtain the region of the intestinal tract investigated here. The region obtained spanned approximately the posterior 3/5 to about the posterior most 1/5 of the intestine. This intestinal portion laid free in the pseudocoelom with no apparent tissue connectivity to the wall of the pseudocoelom, and excludes an anterior 2/5 of the intestine that is embedded in a connective tissue matrix and the posterior-most 1/5 that is fragile and is adherent to the body wall. The regions excluded were the most likely to acquire cell damage during dissection, which could have presented a confounding factor for the analysis. Dissected intestine was placed in 1.5 mL microcentrifuge tubes and was rinsed in PBS then suspended in a 1 mL solution containing 90% fetal bovine serum (F2442, Sigma-Aldrich, St. Louis, MO) and 10% DMSO (922,401 JT Baker, Center Valley PA). Following suspension, the solution was removed to just cover the intestinal sample, then samples were flash frozen in liquid nitrogen, and stored at -80 °C. Samples were shipped on dry ice to Washington University where nuclei were extracted for single cell RNAseq analysis.

### Isolation of nuclei from flash frozen *Ascaris suum* intestine

Prior to extracting nuclei, we assessed numbers of nuclei that comprise the *A. suum* intestine. This was accomplished by staining freshly dissected intestine with the non-vital stain bisbenzimide (Hoechst nuclear dye 33,258, Sigma-Aldrich, St. Louis MO) at 10 µg/mL in PBS for 30 min. Rinsed tissue placed on a glass slide. The intestine assumed a flattened shape, rather than round. It was then viewed using a Nikon Diaphot 300 inverted microscope equipped with epifluorescence capabilities (UV-2 A filter (blue) and photographed with a Nikon D5100 digital camera. Digital images were evaluated by counting the average number of nuclei contained within 50µM × 50µM areas of intestinal tissue, and extrapolating the cell counts to the full intestine.

To obtain single nuclei preparations, flash frozen *A. suum* intestinal tissue (∼ 50 mg) was homogenized in a Dounce homogenizer in 3 mL Lysis Buffer (10 mM Tris-HCl, pH 7.4, 10 mM NaCl, 3 mM MgCl_2_, 0.025% NP-40, and 0.04 U/µL RNasin (Promega)) and incubated on ice 15 min. The suspension was filtered through a 30 μm filter to remove debris and pelleted at 500 × g for 5 min at 4^o^C. Nuclei were washed and filtered twice with Nuclei Wash (1% BSA in PBS with 0.2 U/µL RNasin (Promega)). The nuclei pellets were resuspended in Nuclei Wash at 1000 nuclei / µL and filtered through a 40 μm FlowMi Cell Strainer.

### Responses to drug treatments

To investigate single cell intestinal gene responses to toxic treatments, adult *A. suum* were treated using a protocol employed in other contexts [36]. Briefly, the method involved injection of treatments through the body wall of adult *A. suum*, midway down the length, and into the pseudocoelomic space using a 28-gauge needle. Accuracy of injections was monitored under a dissecting microscope. The volumes of injected solutions were adjusted to 1% of weight of recipient worms, such that the diluent (DMSO) did not exceed 1% of the body weight and final concentrations of toxic treatments were 500 µM CID 1067700 (SML054) or Leflunomide (L5025), (Sigma-Aldrich, St. Louis, MO), based on previous applications with these nematode intestinal toxins/toxicants (NITs [6, 7]). “CID” stands for the “compound identification” number used on PubChem [37], as described in the original study identifying it [38]. Control worms received DMSO only. Two adult female worms injected for each treatment were next incubated for 2 h in PBS (37^o^C) under ambient air conditions. Intestinal samples were then obtained by dissection and processed for scRNA-seq as described above for untreated worms and shipped to Washington University for single cell nuclei preparations.

### Single-Nucleus RNA-Seq sequencing and analysis

Isolated nuclei were subjected to droplet-based 5’ end massively parallel single-cell RNA sequencing using Chromium Single Cell 5’ Reagent Kits as per manufacturer’s instructions (10x Genomics). The libraries were sequenced using a NovaSeq 6000 instrument (Illumina), yielding median 553 million reads and 8104 cells (Supplementary Table S1) detected by the Cell Ranger pipeline (10X Genomics Cell Ranger 3.1.0) using default parameters. While the fraction reads in cells was low (29–43%), the data were sufficient to identify a median of > 200 genes per cell, covering at least 11,800 *A. suum* genes over the whole tissue sample, which represents 70% of all protein coding genes in the *A. suum* genome [39]. We obtained an average of 6,078 nuclei per sample. The data were analyzed using the Seurat package (v4.1.0) [40, 41] available for R statistical analysis environment [42].

The raw read files are accessible on the NCBI Sequence Read Archive (SRA [43], BioProject PRJNA167264), with accession numbers and statistical details per sample provided in Supplementary Table S1. Complete sample metadata, read counts, normalized expression values, and differential expression statistics are provided in Supplementary Table S2. Gene lists per cluster (results of differential expression analysis) are provided in Supplementary Table S3. Biological replicates (numbers shown in Table 1, details shown in Supplementary Table S1) were combined bioinformatically to provide more total cells for more robust downstream analysis.

*C. elegans* L2-stage scRNAseq data from a previous study [5] was downloaded and integrated with untreated *A. suum* sample data. The orthologs between *A. suum* and *C. elegans* were identified using best bidirectional Blastp [44] hits between the predicted proteomes at an E-value threshold of 10^− 5^. Since this set of orthologous genes was much smaller than the whole genome (6,915 genes), the data was filtered to only use genes detected in at least 3 cells, and to only include cells with at least 50 genes detected (i.e. min.cells = 3, min.features = 50). SCTransform [45] (part of Seurat, v4.1.0) was used for normalization and variance stabilization of each sample. A total of 2000 features were used as integration anchors, and the *C. elegans* scRNAseq data [5] was used as integration reference (resolution 0.4).

Potential doublets were identified using doubletFinder v3 [46] with estimated parameters pN = 0.25, pK = 0.05 and an expected doublet rate of 7.5%, yielding 1695 potential doublets. The clustering used for doublet detection was performed using a resolution of 0.4. These potential doublet cells were then removed from their respective samples and the sample data normalized afresh for downstream analysis.

Although not expected due to the use of frozen single nuclei for sequencing, filtering of nuclei based on mapping to mitochondrial genes was performed for all samples. First, since mitochondrial genes are not directly annotated in the current *A. suum* genome annotation, we identified seven putative A. suum mitochondrial genes based on orthology to *C. elegans* mitochondrial genes (AgR009X_g325, COX1; AgR022_g083, COX1; AgR009X_g327, COX3; AgE51_g007, ND1; AgR009X_g326, ND4; AgE51_g006, ND5; and AgB02_g498, ND6). Since these are likely to be only part of the full set of mitochondrial genes in *A. suum*, and also because the expected rate of mapping to these mitochondrial genes in a valid nuclear barcode is much less than the rate usually observed in valid cells in a single-cell sequencing experiment, we used a relatively low “percent.mt” threshold for filtering barcodes (percent.mt < = 1). As a result, cells exceeding 1% mitochondrial transcripts were removed from the analysis.

Potential proliferating cells were identified using Seurat’s ‘cellcyclescoring’ function [41]. *A. suum* orthologs of human S-phase and G2/M-phase genes [47] were identified using best bidirectional Blastp [44] hits between the two predicted proteomes at an E-value threshold of 10^− 5^ (Supplementary Table S4). To adjust the data for differences in cell cycle phase, the normalized data of each sample was rescaled by regressing out the cell-cycle signal (specifically, regressing out the difference “S.Score - G2M.Score”), and the samples are then integrated.

The integrated data was clustered at three separate resolutions (0.04, 0.1, 0.4), resulting in three sets of clusters (2, 3, and 7 clusters, respectively). Differentially expressed genes (DEGs) within any two cell populations/clusters were identified using MAST [48] (in Seurat, v4.1.0). For this, avg_logFC (average of log fold-change) threshold of 0.25 and *p*-value threshold of 0.01 (FDR-adjusted) were used, and genes that aren’t detected in at least 10% cells of the population with high expression were removed. For all clustering, 30 Principal Components (PCs) were used.

To transfer the annotation from untreated samples clustering to drug treatment samples, the label transfer process of Seurat was used, using untreated sample clustering as reference, and drug treatment samples as query. The cluster association with drug treatment was analyzed after normalizing each NIT + DMSO integrated dataset to 10,000 cells per treatment. Enrichment of association with each cluster was determined using a Chi-squared test.

### Functional annotation database

Functional annotations were assigned to all *A. suum* genes in the current genome annotation downloaded from WormBase Parasite [49] (PRJNA62057) using (i) Sma3s (version 2) [50], (ii) PANNZER (2022 release) [51], (iii) KEGG gene annotations [52] using GhostKOALA v2.2 [53], (iv) results from InterProScan v5.42 [54] to identify InterPro functional domains [55] and associated gene ontology classifications [56], (v) orthologs of *C. elegans* genes based on bi-directional best Blastp hits [44] (BLAST, version v2.13.0+, default settings). Proteins with no matches to any of these functional annotation approaches were considered to be “unannotated” (5,647 genes, 33.7% of all genes). Potentially secreted proteins were identified using both SignalP v5.0 [57] for signal peptides and transmembrane domains. For that analysis, proteins with fewer than 2 TM domains and a predicted signal peptide were annotated as having a signal peptide, and proteins with 2 or more transmembrane domains were considered to be transmembrane proteins.

Protein conservation data across nematodes and hosts was quantified using BLAST [44] (version 2.13.0+) and OrthoFinder (v2.4.1) [58], used to compare protein sequences across 22 species and define orthologous protein families (OPFs), using default values. Protein sequence data was downloaded from WormBase Parasite [49] (WBPS15 WS276) for *Ascaris suum* (PRJNA62057), *Ascaris lumbricoides* (PRJEB4950), *Brugia malayi* (PRJNA10729), *Wuchereria bancrofti* (PRJNA275548), *Loa loa* (PRJNA246086), *Dirofilaria immitis* (PRJEB1797), *Trichuris muris* (PRJEB126), *Trichuris suis* (PRJNA179528), *Trichuris trichiura* (PRJEB535), *Strongyloides ratti* (PRJEB125), *Strongyloides stercoralis* (PRJEB528), *Ancylostoma ceylanicum* (PRJNA72583), *Necator americanus* (PRJNA72135), *Haemonchus contortus* (PRJEB506) and *Caenorhabditis elegans* (PRJNA13758). In addition, protein sequence data for hosts and outgroups were downloaded from Ensembl [59] for *Homo sapiens* (Human; GRCh38.p13), *Bos taurus* (ARS-UCD1.2), *Sus scrofa* (Sscrofa11.1), *Ovis aries* (Oar_v3.1), *Mus musculus* (GRCh38), *Drosophila Melanogaster* (BDGP6.32) and *Saccharomyces cerevisiae* (R64-1-1). The top BLAST [44] hit for each *A. suum* protein was also identified, including the E value, alignment length, % identity, and whether the top hit was reciprocal (NCBI blastp v2.13.1+, default settings).

High-throughput transcriptomic and proteomic datasets from previous studies were remapped to the current *A. suum* genome annotation (PRJNA62057 [39, 49]), to enable comparisons between genes identified in the new single cell dataset and existing datasets. Raw RNA-seq reads from various *A. suum* tissues (intestine, pharynx, and head from adult male and female worms, and ovary, uterus, testis and seminal vesicles) that were previously analyzed [60] were downloaded and remapped to the *A. suum* assembly using STAR v2.7.5b [61] and reads per gene were quantified with featureCounts (from Subread package 2.0.3) [62]. Normalized gene expression levels (FPKM) were calculated per gene per sample. Intestine-overexpressed genes were identified as any genes with non-zero expression in all four intestine samples, and a minimum 5-fold high average expression level in the intestine relative to the average expression level of all of the other tissues (735 genes). Raw proteomics data from five *A. suum* intestine samples from a previous analysis [63] (pseudocoelomic fluid, intestinal lumen PBS wash, intestinal lumen 4 M urea wash, intestine tissue homogenate, and intestine tissue homogenate that forms a pellet after centrifugation between 5,000 and 50,000 g) were downloaded and MaxQuant [64] (version 2.4.2.0) was used to match and quantify MS/MS spectra to predicted *A. suum* peptides. Peptides with a sequence match in pigs were removed, and then the number of peptide matches was quantified for each protein in each sample. Proteins were considered to be detected in an intestinal sample if they were identified with 2 or more peptides and were not detected in the pseudocoelomic fluid sample. Additionally, genes differentially expressed by Leflunomide and CID 1067700 in *A. suum* L3-stage larvae were identified based on previous results [7].

All functional annotations and re-processed data from previous studies, for all genes, are available in Supplementary Table S2.

### Functional enrichment testing

Functional term / pathway enrichment among DEGs was determined using Gostats v2.50 [65] for gene ontology terms (performed in R) and WebGestalt v2019 [66] for InterPro domains and KEGG pathways (performed on the WebGestalt website, https://www.webgestalt.org/). Both approaches used over-representation analysis (ORA) comparing gene sets of interest vs. a background of all detected genes across all samples, with an FDR-adjusted *P* ≤ 0.05, and a minimum 3 genes of interest being required for each pathway / term.

### Statistics and reproducibility

Additional enrichment tests were ran on gene sets of interest using negative binomial distribution tests (performed using MS Excel v16.77), to test enrichment based on annotations described above, including: (i) genes with functional annotations (i.e., not “unannotated” as described above), (ii) genes with signal peptides, (iii) transmembrane domain proteins, (iv) adult *A. suum* intestine-overexpressed genes [60], (v) proteomic detection in one of the four adult *A. suum* intestinal proteomics samples [63], (vi) L3-stage *A. suum* Leflunomide and CID 1067700 upregulated and downregulated genes, after 1 and 2 h [7] and (vii) conservation data based on OrthoFinder results, including genes with orthologs exclusively in *A. suum*, *Ascaris* (*A. suum* and *A. lumbricoides*), Clade III nematodes (*A. lumbricoides, Ascaris suum*, *Brugia malayi*, *Wuchereria bancrofti*, *Loa loa* and/or *Dirofilaria immitis*) or all nematodes, as well as genes conserved among all clade III species, all nematodes, or all species. Statistical testing was performed for all of these comparisons whenever the overlap between a gene set of interest and one of the annotations was greater than 3, then FDR correction was performed to multiple test correction for *P* values across all comparisons.

All R scripts used to generate results have been made publicly available for download on GitHub: https://github.com/brucearosa/Asuum_Intestine_scRNAseq.

## Results and discussion

**Fig. 1:**
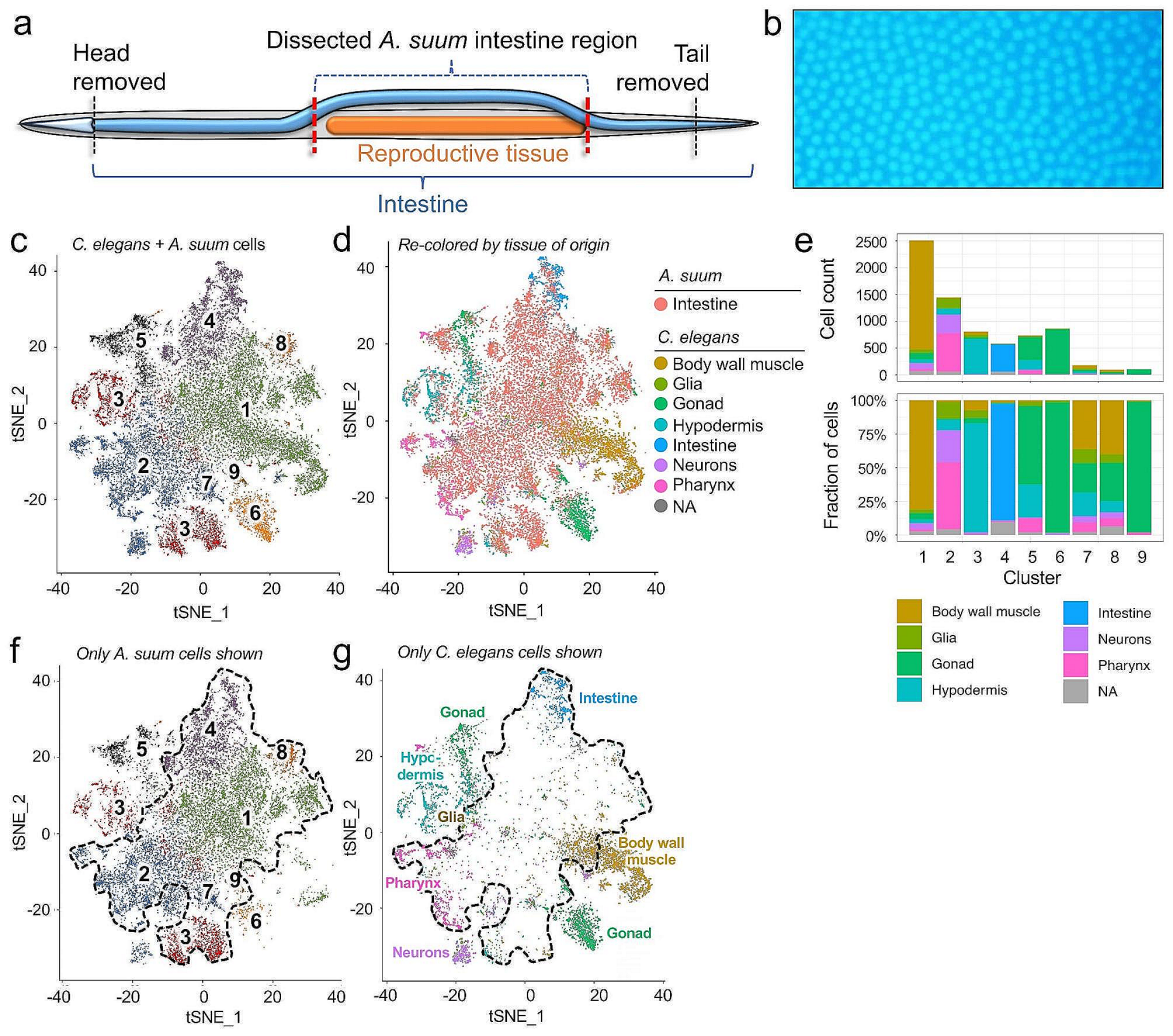
Clustering of ***A. suum*intestinal cells relative to*C. elegans*L2 whole-worm cells. a**. A schematic describing the intestinal region comprising the ∼ 2/5th s of the whole intestinal length that was dissected for scRNAseq analysis. **b.** Bisbenzimide-stained adult female *A. suum* intestine image used to estimate total cell count per intestine. **c.** tSNE visualization of integrated single-cell data from *A. suum* untreated samples and the previously published single-cell *C. elegans* L2 whole worm sample, colored according to assigned cluster numbers. **d**. The same tSNE plot, recolored to instead distinguish *A. suum* intestine cells (red) from *C. elegans* cells, colored according to previously defined source tissues. **e**. The number and fraction of *C. elegans* cells in each cluster, colored by previously defined *C. elegans* tissue annotation. **f**. The same tSNE clustering showing only *A. suum* intestinal cells. The black border highlights the region containing clusters enriched in *C. elegans* intestine or not assigned (NA) cells, and 72.7% of all *A. suum* intestine cells. **g**. The same tSNE clustering showing only *C. elegans* whole L2 cells, labeled according to previously defined source tissues

The intestinal segment we investigated (hereinafter referred to as the intestine) comprises approximately 2/5th of the entire intestinal length and originated from a region of the intestine that lies free in the pseudocoelom with apparently no tissue connectivity to the wall of the pseudocoelom (*see* Methods) and thus provides an initial view of functional heterogeneity of this tissue (Fig. 1a). In an adult female *A. suum* with a single cell-layer intestine of 10 cm length and 0.5 cm width, our calculation based on bisbenzimide-stained intestinal cells indicates there are approximately 8,266,800 cells per intestine (Fig. 1b; Supplementary Fig. S1A).

To assess cellular functional heterogeneity in the parasitic nematode intestine, we carried out single-cell transcriptomic analysis on 9 *A. suum* intestinal tissue samples, comprising 4 sets of treatments: untreated (*n* = 3; to establish a baseline cell transcriptional state), vehicle-treated (DMSO; *n* = 2), and treatment with 2 different nematode intestinal toxins (NITs; *n* = 2 each for CID 1067700 and Leflunomide; Table 1; Supplementary Table S1). These NITs were selected based on previous studies that demonstrated that they cause intestinal cell and tissue destruction in *A. suum* [6, 7], ensuring a robust phenotype and transcriptomic response in the intestinal cells, as opposed to drugs which kill worms via other mechanisms. We first compared *A. suum* single intestinal cell transcriptomes from untreated parasites with an existing *C. elegans* scRNAseq whole-worm dataset, followed by resolution of single intestinal cell transcriptome diversity within *A. suum*. We next analyzed gene transcriptional responsiveness among single *A. suum* intestinal cells following treatment with known NITs. The overall scRNAseq workflow is described in Supplementary Fig. S2. Data per gene including complete functional annotations, differential expression statistics, normalized average expression levels, and data reanalyzed from previous studies (see methods) are provided in Supplementary Table S2. Note that nuclei from single cells were extracted and sequenced for the analysis; Throughout the results/discussion, we will refer to this as “single-cell” sequencing for the sake of consistency.

**Table 1:** Sample statistics from Cell Ranger, and after quality control and filtering. UMI = unique molecular identifier. Values represent the average value or the sum of values (bold) of the replicates. Complete data per replicate including accessions are provided in Supplementary Table S1

Treatment cohort	Untreated	DMSO-treated	CID 1067700-treated	Leflunomide-treated
Number of replicates	3	2	2	2
Cell Ranger output				
**Estimated number of cells**	26,424	10,925	6,325	11,030
**Number of reads (million)**	1,710.1	978	898	1,074
Mean reads per cell	66,852	91,517	145,922	106,861
Median genes per cell	391.7	690.5	879.5	562.5
Fraction of reads in cells	39.9%	30.8%	37.3%	40.5%
Total genes detected	14,024	13,832	13,501	13,686
Median UMI counts per cell	2,516	3,883	6,449	3,908
Filtered statistics				
**Number of cells**	15,129	10,924	6,325	10,952
Mean UMIs per cell	3,650	5,580	8,411	6,596
Median genes per cell	495	971	1,197	952

### Novel transcriptional diversity among intestinal cell populations in *A. suum* compared to *C. elegans*

In *A. suum*, untreated intestinal cells a median detection of 12,192 genes was recorded, which is 72.7% of all protein coding genes in the *A. suum* genome [39]. Simple clustering of this transcriptional data will resolve cells into clusters, but in the absence of any validation of clustering resolution, it is not clear what the biological significance of these clusters might be. Hence, we chose to gain initial validation of biological significance, followed by result-guided clustering of the *A. suum* intestinal cell data. To this end, phenotypic links with *C. elegans* intestinal cells were sought for *A. suum* intestinal cells using previously published data from a single cell transcriptome analysis of whole *C. elegans* L2 larvae [5]. These data were integrated with data from our three *A. suum* untreated intestinal samples (using 6,915 one-to-one bidirectional best BLAST hits; Supplementary Table S2). For the *C. elegans* dataset, only the previous “experiment 2” data were integrated, since only that experiment included data from intestinal cells [5]. For this analysis, 15,114 *A. suum* cells and 7,325 *C. elegans* cells remained after filtering. A clustering resolution of 0.4 corresponding to nine clusters (Fig. 1c) was selected because the known *C. elegans* tissues assigned in the previous study [5] (Fig. 1d) largely comprise separate clusters, indicating strong agreement between the integrated results and the previously published results from *C. elegans* [5], despite the inclusion of twice as many *A. suum* intestinal cells. This relative homogeneity for designated tissue clusters is shown in Fig. 1e, where among clusters with > 500 *C. elegans* cells, only gonad cells are substantially distributed over two clusters (Clusters 5 and 6), with other tissues highly associated with a single large cluster (hypodermis with Cluster 3, intestine with Cluster 4 etc.). Such separation is consistent with tissue-based clustering reported in Cao et al. [5] and suggests that for this data at this resolution, large *C. elegans* clusters represent cellular heterogeneity that is biologically relevant at the organ level. On the other hand, clustering at a resolution higher than biologically relevant would have broken single tissue clusters into multiple subclusters. Thus, we determined that this clustering resolution provides a valid baseline to gain insight on comparative functional heterogeneity of *A. suum* intestinal cells.

Comparing cells comprising these integrated clusters, we note that in Fig. 1c, Cluster 4 (containing the intestinal cluster among *C. elegans* cells) has two subregions, with *C. elegans* intestinal cells mostly restricted to the upper subregion. The *A. suum* intestinal cells resolved in Cluster 4 (Fig. 1f) comprise at least two subsets, one that shows greatest congruence of transcriptional phenotype to *C. elegans* intestinal cells and another that shows moderate congruence with *C. elegans* intestinal cells. Nevertheless, 85% of *A. suum* intestinal cells localized to clusters other than Cluster 4, with Cluster 1 containing 35% of all *A. suum* intestinal cells and largely occupying space in the cluster map where a small number of scattered cells from *C. elegans* lack confident organ assignment (Fig. 1f). Although one subregion of Cluster 1 overlays body wall muscle cells from *C. elegans*, this subregion is largely devoid of Cluster 1 *A. suum* cells (Fig. 1g). Consequently, *A. suum* intestinal cells in Cluster 1 exhibit distinct transcriptional phenotype(s) comparable to differences between different tissues at the resolution set for this analysis. Further functional assessment of the *A. suum* intestinal cell clusters is described below.

The area of Cluster 4 combined with the area for which there is no *C. elegans*-confident organ assignment is circumscribed in Fig. 1f, with 79.2% of *A. suum* cells mapping to this region. The remaining approximately 20% of *A. suum* intestinal cells showed some transcriptional congruence to other *C. elegans* organ types spread primarily across Clusters 1, 3, 5 and 6, which include *C. elegans* cells primarily assigned to body wall musculature, hypodermis, gonad (cluster 5) and gonad (cluster 6), respectively (Fig. 1g). Based on the isolation procedure, contamination of the *A. suum* intestinal cell preparation with cells from other *A. suum* organs is not likely to account for these associations. Rather, the weak associations detected may result from the parameters needed to sort *C. elegans* organs when scRNAseq data from both species are clustered together, or perhaps a low gene sample size for the given *A. suum* cells, or a combination of both effects.

Beyond establishing methods to discern thresholds of biological relevance using cross species comparisons, results from this integration of *A. suum* and *C. elegans* data has implications for studying evolution of the nematode intestine. The observed conservation in single cell intestinal gene and transcript phenotypes between phylogenetically distant species (ca. 350 mya for *A. suum* and *C. elegans* [67]) should support the derivation of widely conserved intestinal cell gene expression across the Nematoda. Additionally, the marked interspecies differences detected for major intestinal cell populations of *A. suum* may indicate adaptations of importance for successful parasitism of this species. For instance, it is conceivable that the large number of cells comprising the *A. suum* intestine has facilitated divergence and specialization of intestinal cell subsets not uniformly represented across nematode species. More interspecies comparisons using scRNA may be informative on this point. A caveat to this discussion is that the *C. elegans* cells involved in the analysis were derived from a larval L2 of this species, which might lack single cell phenotypes associated with adult worms of this species, which will also require further study. It should also be noted that the term “cell populations” is used to refer to transcriptionally distinct intestinal cells, with no intended implication that these necessarily represent biologically distinct terminally differentiated cells, cells at different stages of development, or similar cells undergoing different transcriptional phases. These possibilities will be explored in more detail in the sections below.

Overall, the integrated clustering results support that at clustering resolutions resulting in distinct tissue clusters in *C. elegans*, we observe real biological heterogeneity among *A. suum* intestinal cells. Further, the clustering method utilized here has proven capable to better define functionally distinct cellular populations that comprise the *A. suum* intestine, which will be investigated below.

### Intestine of adult *A. suum* consists of at least three main cell populations

**Fig. 2:**
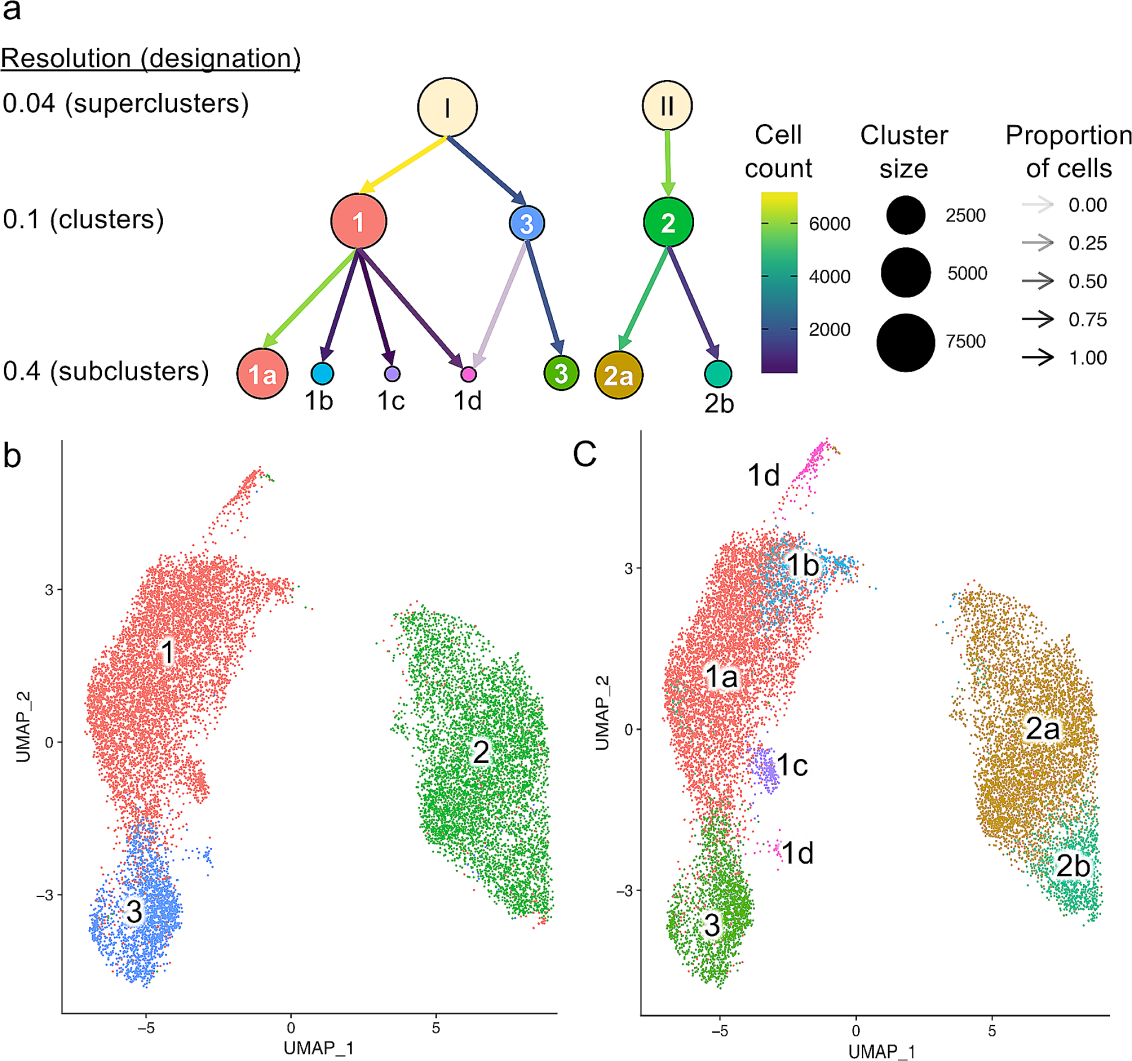
Subpopulations of major cell clusters. a. Minor subpopulations separate out from the major clusters, as clustering is done at increasingly higher resolutions. This leads to 4 small populations being identified: clusters 2b, 1b, 1c and 1d. Arrow colors and opacity indicate the number and the proportion of cells assigned to each subcluster from each larger cluster (respectively). **b**. UMAP visualization showing Clusters 1–3. **c**. Smaller subpopulations of major clusters shown on the same UMAP layout as in panel **b**

The untreated *A. suum* intestinal scRNA datasets were next integrated and clustered, absent *C. elegans* data, using 12,169 cells after filtering. Clustering was performed at three separate resolutions (0.04, 0.1, 0.4), each of which yielded different numbers of clusters (ranging from 2 to 7) (Fig. 2a): (i) The lowest resolution (0.04) clustering shows 2 major transcriptional profiles (superclusters I and II), with no overlap in cellular membership even at higher resolutions. This cluster resolution was strongly supported by observing the clustering image (Fig. 2b), which clearly shows two very distinct clusters; (ii) A third cell population emerges out of supercluster I cells at the next higher resolution resulting in 3 clusters (0.1, blue cluster in Fig. 2b) and hereinafter referred to 3-cluster model (3-CM). This 3-CM resolution is also well-supported by the overall clustering, with supercluster I visually demonstrating two distinct major clusters; (iii) Clustering at the highest resolution (0.4, which was guided by the *C. elegans* comparison presented in Fig. 1) resolved 7 subclusters (7-cluster model; 7-CM). These 7 subclusters include 4 subclusters of cluster I (the large subcluster 1a and smaller subclusters 1b, 1c, and 1d), 2 subclusters of cluster 2 (the large subcluster 2a and a smaller subcluster 2b; Fig. 2c), and cluster 3 which showed no further subclustering. At this 7-cluster resolution the 4 largest subclusters (1a, 2a, 2b, and 3) account for > 93% of all *A. suum* intestinal cells analyzed.

Of the 3 different *A. suum* clustering models defined in this series of resolution analyses, the 3- cluster model (3-CM), resolved at the 0.1 resolution setting, has features that make it most attractive for further discussions. First, this model was derived below the 0.4 setting used in the *C. elegans* comparison, reducing the possibility of over-resolution of clusters. Second, by comparison to *A. suum* clusters 1 and 2, cluster 3 was identified at this resolution level, and *A. suum* cells in this cluster most strongly resemble the *C. elegans* intestinal cells identified in Cao et al. [5] based on canonical intestinal markers (Supplementary Fig. S3). In contrast, cluster 1 in this model shows an intermediate resemblance to *C. elegans* intestinal cells and cluster 2 shows marked differences with *C. elegans* intestinal cells (Supplementary Fig. S4a). Third, additional subclusters of *A. suum* intestinal cells obtained at the 0.4 setting in the 7-CM were generally comprised of smaller numbers of cells and showed relatively diffuse distribution (Supplementary Fig. S4b-e) overlapping larger clusters 1 and 2 with transcriptional profiles that lacked confident assignment to any annotated cell type of *C. elegans* (clusters 1 and 2 regions in Supplementary Fig. 4a compared to Fig. 1g). Thus, it is likely that some, or all, of these additional subclusters result from over resolution, and not contamination from other tissues. Consequently, we will focus further discussion on the 3-CM, while acknowledging that the 7-CM may convey biological significance deserving further investigation.

**Fig. 3:**
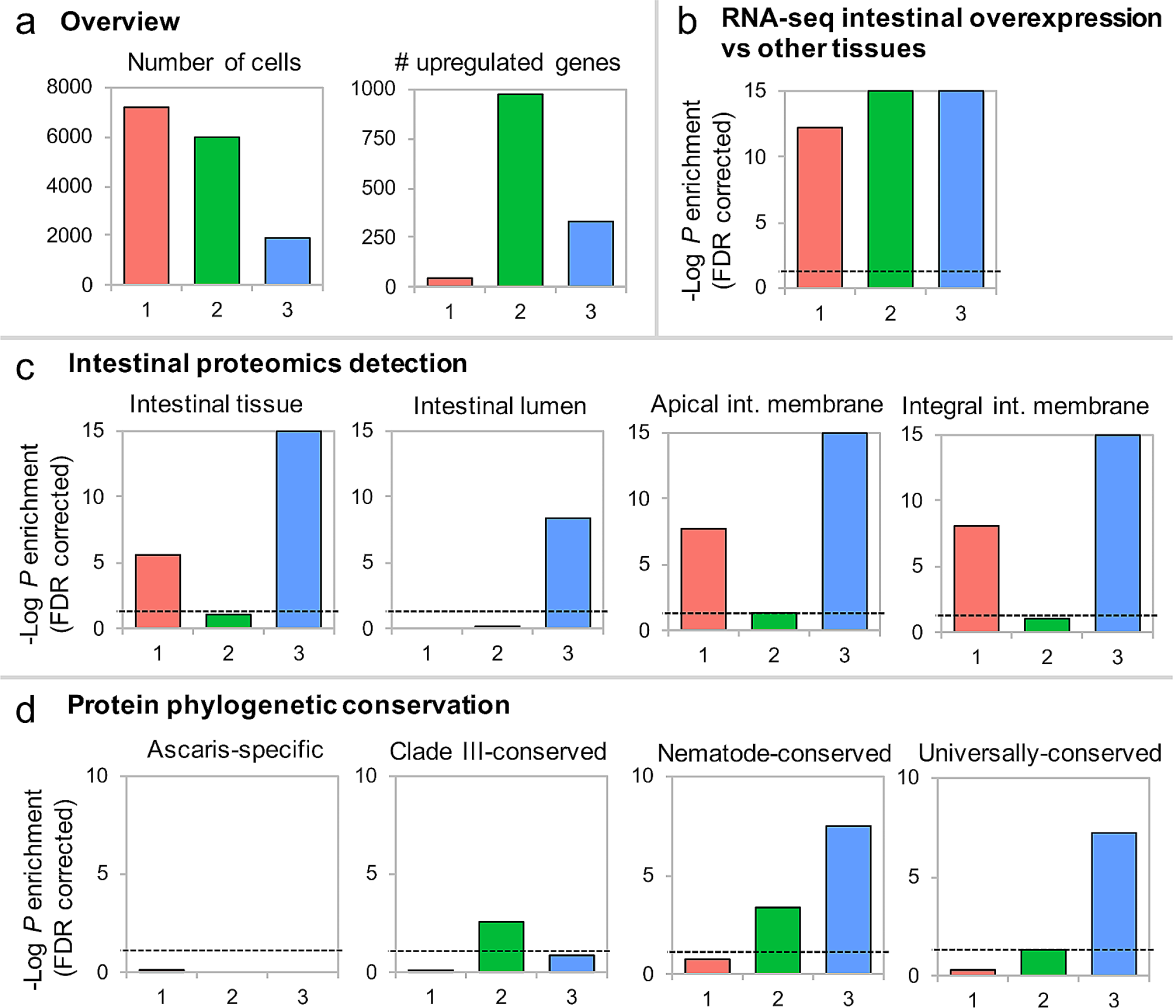
Characteristic of major cell clusters (1, 2, and 3) and comparison with previously published results. a. Cluster overview, size (left) and number of DE genes (right). **b**. Enrichment of previously published intestine-overexpressed genes (Rosa et al., 2014). **c**. Enrichment of genes previously detected in intestinal proteome (Rosa et al., 2015). **d**. Enrichment of genes conserved at different levels of monophyletic groups. All enrichment tests were performed with binomial distribution tests and FDR-corrected for the number of tests performed

Previously established omics databases for the *A. suum* intestine were next used to gauge if differences documentable among *A. suum* clusters 1, 2 and 3 support validity of the 3-CM. The number of cells contained in the clusters ranged from high to low; cluster 1 (7,262 cells, 48% of all cells) > cluster 2 (5,964 cells, 39.4%) > cluster 3 (1,903 cells, 12.6%; Fig. 3a), indicating that clusters 1 and 2, which each showed less similarity to *C. elegans* intestinal cells, had higher cell representation than cluster 3, which displayed most similarity to *C. elegans* intestinal cells. Conversely, cluster 2 expressed the highest number of intestine-overexpressed genes among the 3 clusters, followed by cluster 3 and then 1 (Fig. 3a), based on a differential expression analysis among 10 *Ascaris suum* tissues including the adult male and female intestine [60]. Nevertheless, all the clusters show enrichment for genes relatively overexpressed in the intestine, as compared to *A. suum* non-intestinal tissues (Fig. 3b) [60]. Mass-spectrometry based proteomics data [63] provided more specific comparisons and showed that only cluster 3-overexpressed genes were significantly enriched for proteins detected from intestinal lumen (*P* = 2.2 × 10^− 9^; FDR-corrected binomial distribution test), while clusters 1 and 3, but not 2, had significant enrichment for proteins detected in the intestinal tissue, apical intestinal membrane and integral intestinal membrane (*P* < 10^− 5^ for all comparisons; Fig. 3c). Cluster 3 showed higher enrichment in each of these protein categories compared to cluster 1. In contrast, cluster 2 was the only cluster enriched for genes more likely to be conserved across Clade III nematode species, and cluster 3 was enriched for genes that were conserved more widely (among phylum Nematoda, or universally conserved among nematodes and mammal hosts; see OrthoFinder [58] in methods), especially compared to cluster 1 (Fig. 3d).

Overall, *A. suum* omics-based comparisons are consistent with the assertion that *A. suum* cluster 3 intestinal cells are more closely related to the *C. elegans* intestinal cells compared to clusters 1 and 2 (Supplementary Fig. S4a). Moreover, certain genes previously used as *C. elegans* intestinal markers (nep-17 [5, 68]) or intestine-enriched (vit-6 [68]) were observed to have cluster 3-specific or cluster 3-enriched expression (Supplementary Fig. S3). The top 10 most significantly overexpressed genes in each of the three clusters according to MAST [48] differential expression analysis are shown in Table 3.

At the other end of the spectrum, *A. suum* cluster 2 is most different from clusters 1 and 3, and from the *C. elegans* intestinal cell phenotype(s). Although it shares intestinal enriched genes (compared to other *A. suum* tissues) with clusters 1 and 3, many proteins that define key intestinal cell compartments (lumen, apical intestinal membrane, etc.) are relatively underrepresented in cluster 2. While the proteomic data set is not exhaustive, it is expected to represent the more abundant intestinal proteins that functionally distinguish the intestine from other tissues [63]. Thus, cluster 2 cells appear to be unique in that they are diminished in features expected of more classical intestinal cell phenotype(s) e.g. clusters 1 and 3. Concurrently, cluster 2 cells show greater enrichment for Clade III-associated genes, supporting the possibility that cluster 2 represents a specialized differentiated intestinal cell phenotype that has evolved within clade III nematodes [69]. This possibility is considered further below.

Cluster 1 cells display greater similarity to cluster 3 cells, given that both clusters 1 and 3 arose from supercluster I (Fig. 2), and cluster 1 cells show intermediate similarity to *C. elegans* intestinal cells by comparison to cluster 3. Transcripts for a significant, but intermediate, representation of *A. suum* intestinal protein genes were detected in cluster 1 cells compared to cluster 3 supporting quantitative differences with cluster 3, inclusive of large differences with respect to nematode specific genes and universally conserved genes (Fig. 3d). Proteins corresponding to the genes overexpressed in cluster 1 and cluster 3 were significantly enriched for signal peptides for secretion (*P* = 8.5 × 10^− 5^ and 7.3 × 10^− 5^, respectively), suggesting that both clusters were enriched for putatively secreted proteins, while cluster 2 was significantly depleted for signal peptides (*P* = 3.2 × 10^− 3^). However, cluster 1 was the only cluster with overexpressed genes that were not significantly enriched for functional annotations (*P* = 0.27), suggesting that it represents a novel set of secreted proteins with no currently known function. These comparisons support that clusters 1 and 3 represent separate cell phenotypes.

The likelihood for existence of multiple intestinal cell phenotypes is high in the *A. suum* intestine given the gene transcript expression profiles that differ in tissue obtained along its length [4]. However, within this dataset, no enrichment was identified among any cluster or subcluster with genes overexpressed in the anterior, middle or posterior of the intestine based on that previous study (FDR-corrected binomial distribution test) [4], indicating that transcriptional differences between these novel intestinal cell types are not associated with known transcriptional differences along the length of the intestine. Single cell gene expression differences that distinguish *A. suum* intestinal clusters 1, 2 and 3 in the 3-CM establish previously unappreciated criteria that arguably define at least 3 major cell phenotypes comprising the segment of the intestine investigated here.

### Major cell clusters reflect distinct functional pathway enrichment

**Fig. 4:**
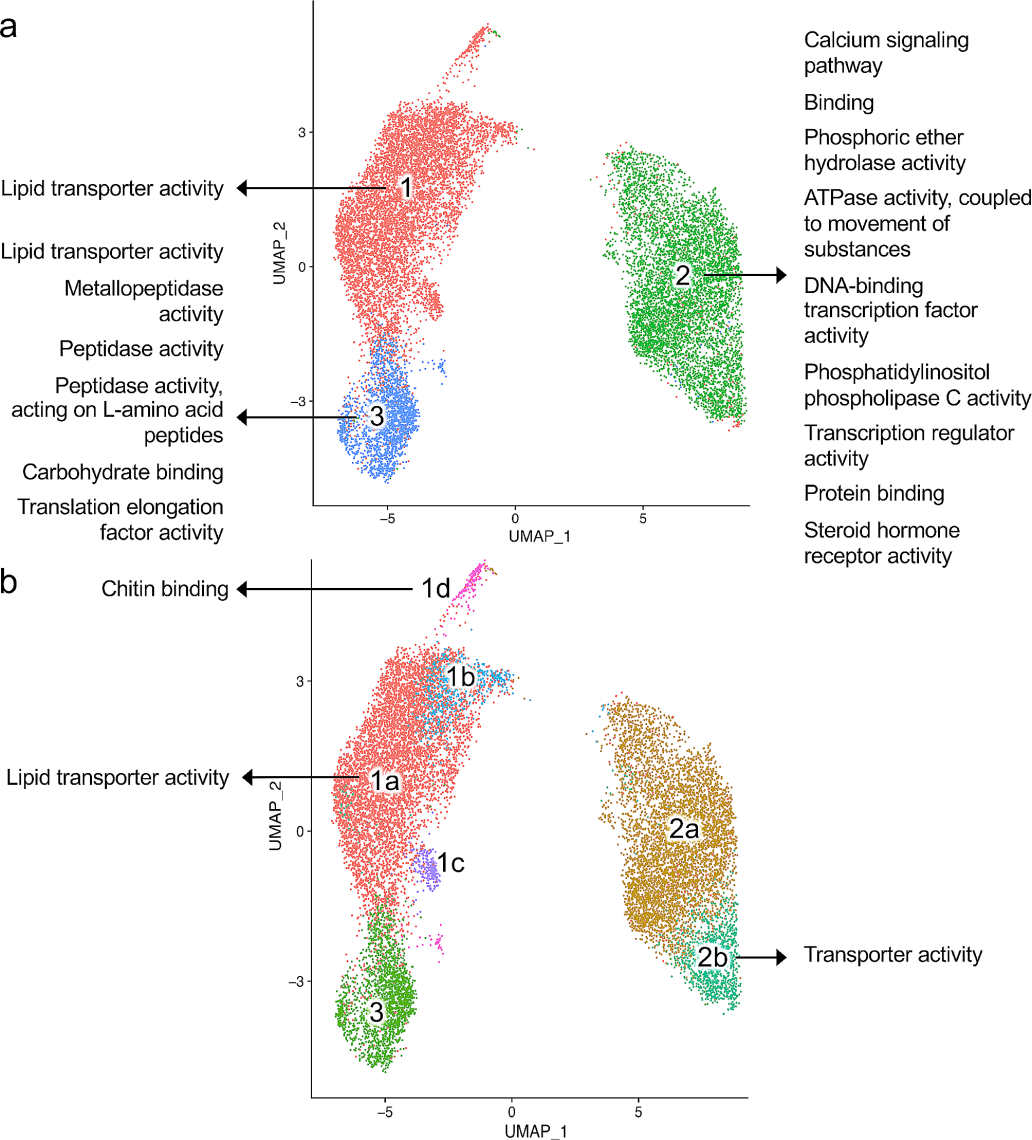
Gene Ontology molecular function terms enriched in subpopulations of cells (a) Functions enriched in the cells belonging to the 3 major clusters. (**b**) Functions enriched among the minor subpopulations of the major clusters, relative to other subclusters. Some genes but no functions were significantly enriched for 1b or 1c, but gene lists can be found in Supplementary Table S3. No genes were significantly higher in subcluster 2a compared to 2b. All Gene Ontology enrichment testing was performed using GOstats (FDR-corrected *P* values for enrichment, min. 3 enriched genes per category)

To better assess the functional heterogeneity that distinguishes each of the 3 clusters in the *A. suum* 3-cluster intestinal cell model we identified marker genes for each of these clusters, i.e. significantly differentially expressed (DE) genes between each cluster and all the other cells, and also analyzed genes that differentiated cluster 3 from cluster 1. All differential expression statistics for all comparisons and all genes are provided in Supplementary Table S2, and gene lists and function enrichment tables for each comparison are provided in Supplementary Table S3. The DE genes predominantly had higher expression levels in cluster 2 and lower in cluster 1, with only 44 genes having significantly higher expression in cluster 1 (Supplementary Table S3a), compared to 976 in cluster 2 (Table 2; Fig. 4a; Supplementary Table S3b). The functions enriched in genes significantly overexpressed in cluster 2 include those critical to certain signal transduction processes e.g. phosphoinositol phospholipase C (PLC) activity and Calcium Signaling pathway (Fig. 4a). It is notable that 230 out of 976 cluster 2 markers are not functionally annotated (by KEGG [52], InterPro [55] or Gene Ontology [56]), including 186 that are *Ascaris suum*-specific (based on a comparison with 22 species; see OrthoFinder [58] analysis described in methods). Cluster 3-overexpressed genes are enriched in “lipid transporter” and peptidase activities (Fig. 4a, Supplementary Table S3c). In this case, 36 out of 331 overexpressed genes do not have any functional annotation assigned, and 40 were *Ascaris suum*-specific. The 44 cluster 1-overexpressed genes were only enriched in lipid transport processes. The top 10 most significant DE genes in each of the three clusters are shown in Table 3.

**Table 2:** High and low expression genes marking the three primary clusters and the 4 subpopulations of the larger clusters

Cluster / subcluster	Cluster(s) used for comparison	Number of genes All (functionally annotated)	
		Higher	Lower
1	2 and 3	44 (37)	853 (682)
2	1 and 3	976 (780)	195 (174)
3	1 and 2	331 (298)	321 (245)
1b	1a	7 (1)	283 (251)
1c	1a	25 (20)	11 (10)
1d	1a	87 (71)	3 (3)
2b	2a	58 (32)	0 (0)

**Table 3:** The top 10 most significantly overexpressed genes in each cluster, compared to the other clusters

Cluster	Gene ID	Functional annotation	Phylogenetic Conservation	Log_2_ Fold Change	Adjusted *P* value
Cluster 1	AgR002_g271	otud4: Zgc:165,536 protein	Nematode-specific	1.66	0
	AgR049_g076	-	*Ascaris*-specific	0.52	0
	AgR049_g078	-	*Ascaris*-specific	0.50	0
	AgR001_g371	GRANULINS domain-containing protein	*Ascaris*-specific	0.49	0
	AgB23_g001	-	*A. suum*-specific	0.47	0
	AgR028_g116	fabG: DeHydrogenases, Short chain	Nematodes & hosts	0.43	0
	AgB04_g312	eef2: Elongation factor 2	Nematodes & hosts	0.43	0
	AgR207X_g001	clec-160: C-type lectin protein	Nematode-specific	0.43	0
	AgR063_g005	vit-6: Vitellogenin-6	Nematodes & hosts	0.41	0
	AgR159_g003	vit-6: Vitellogenin-6	Nematodes & hosts	0.41	0
Cluster 2	AgB01_g233	-	*A. suum*-specific	2.95	0
	AgB01_g238	-	*A. suum*-specific	2.84	0
	AgR007_g007	-	*Ascaris*-specific	2.69	0
	AgB04_g065	IPR001005: SANT/Myb domain	*Ascaris*-specific	2.54	0
	AgR006_g005	tos-1 (Target Of Splicing)	Nematode-specific	2.42	0
	AgR009X_g305	Transmembrane protein	Ascaris-specific	2.28	0
	AgR039_g009	Endoribonuclease XendoU	Nematodes & hosts	2.16	0
	AgR019X_g186	-	Clade III-specific	2.13	0
	AgB13X_g054	Nuclear factor related to kappa-B-binding	Nematode-specific	2.11	0
	AgB01_g234	-	*A. suum*-specific	2.06	0
Cluster 3	AgR013X_g192	vit-6: Vitellogenin-6	Nematodes & hosts	1.19	0
	AgB02_g451	ABA-1: Polyprotein ABA-1	Nematode-specific	1.15	0
	AgR011_g069	GOLGB1: golgin subfamily B member 1	Nematode-specific	1.11	0
	AgR005_g235	vit-6: Vitellogenin-6	Nematodes & hosts	1.08	0
	AgR011_g070	GOLGB1: golgin subfamily B member 1	*Ascaris*-specific	1.07	0
	AgR015_g155	ASU_02895: Apolipophorin	Nematodes & hosts	1.06	0
	AgR063_g005	vit-6: Vitellogenin-6	Nematodes & hosts	1.04	0
	AgR159_g003	vit-6: Vitellogenin-6	Nematodes & hosts	1.00	0
	AgR001_g371	GRANULINS domain-containing protein	*Ascaris*-specific	0.90	0
	AgR028_g116	fabG: DeHydrogenases, Short chain	Nematodes & hosts	0.89	0

Since *A. suum* cluster 2 shows the greatest phenotypic differences among the three clusters and when compared to *C. elegans* intestinal cells, we analyzed genes that showed highly cluster 2-specific expression (i.e. expressed in > 85% of cells in cluster 2, but no more than 15% of all non-cluster 2 cells). These genes are listed in Table 4, and include many species-specific genes (i.e. with no orthologs found in any other nematode analyzed). PLCE, which is associated with the PLC activity enriched in all cluster 2 markers is also among these cluster 2 exclusive genes.

**Table 4:** Markers of cluster 2 (i.e. DE genes between cluster 2 and all other cells). Detected in > = 85% of cluster 2 cells, but no more than 15% of other cells. N/A

Gene ID	Functional annotation	Phylogenetic Conservation	Log_2_ Fold Change	Proportion of cells	
				In cluster 2	In all other cells
AgR007_g007	-	Ascaris-specific	2.69	99.3%	12.7%
AgR006_g005	Target Of Splicing tos-1	Nematode-specific	2.42	98.5%	14.9%
AgR009X_g305	-	Ascaris-specific	2.28	96.8%	8.5%
AgR019X_g186	-	Clade III-specific	2.13	96.4%	4.9%
AgR003_g101	Phosphoinositide phospholipase C	Nematodes & hosts	1.80	95.6%	9.0%
AgR032_g058	Bifunctional coenzyme A synthase	Nematodes & hosts	1.98	94.4%	5.1%
AgR056_g040	Microtubule-associated protein 1 A, putative	Ascaris-specific	1.99	93.0%	6.4%
AgR001_g395	FBXO6: F-box protein 6	Nematodes & hosts	1.53	90.9%	14.3%
AgB05_g187	DIAPH2: diaphanous 2	Nematodes & hosts	1.46	89.8%	8.0%
AgR009X_g301	Transmembrane protein 184B	Nematodes & hosts	1.82	89.4%	3.5%
AgR003_g312	CNOT3: CCR4-NOT transcription complex subunit 3	Nematodes & hosts	1.51	87.0%	4.4%
AgR090_g001	FERM domain-containing protein 8 (FRMD8)	Nematodes & hosts	1.84	85.4%	7.0%
AgB08X_g090	Receptor protein-tyrosine kinase / EGFR	Nematodes & hosts	1.37	85.1%	7.3%

Since cluster 2 is the most distinct at the 3-CM, with clusters 1 and 3 closer to each other (Fig. 2b), it is interesting to consider the features distinguishing cluster 3 from cluster 1, rather than to all non-cluster 3 cells, which would likely obscure the differences between clusters 1 and 3 because of the larger cluster-2 specific signal. Comparing cluster 3 to cluster 1, 308 genes were overexpressed in cluster 3 (Supplementary Table S3d) and only 2 genes were overexpressed in cluster 1 (Supplementary Table S3e). The two cluster 1-overexpressed genes included one unannotated gene (AgR002_g270) and an ortholog of de-ubiquitinating enzyme otud4 (AgR002_g271), with no previous description in the literature in nematodes. The 308 cluster 3-overexpressed genes are significantly enriched in various peptidase and metabolic processes (GOstats [65]), along with high expression of genes such as Vitellogenin, C-type lectins and lipid transport proteins (InterPro domains enriched according to over-representation testing using WebGestalt [66]; Fig. 4a). Vitellogenin, among the 44 genes that are overexpressed in cluster 1 and showing even higher expression in cluster 3, is a yolk protein, but is known to be synthesized in and secreted from intestine during the egg-laying stage of the female in *C. elegans* [70] and then taken up by the oocytes through endocytosis [71]. *A. suum* has multiple genes annotated as Vit-6 (including four in the top 10 DE genes in cluster 3; Table 3), which have highly consistent expression profiles with respect to each other across clusters and subclusters, leading to vitellogenin function(s) being highly enriched in multiple subpopulations that show relatively high expression of this set of genes (Supplementary Fig. S5a). Based on the previously published *A. suum* bulk RNA-seq tissue analysis [60], these Vit-6 genes are also very highly expressed in both the female and male intestine (Supplementary Fig. S5b), and Tc-vit-6 in the intestinal nematode *Toxocara canis* was also found to have very high gene expression, corresponding with the highest detection by immunohistochemistry in the intestine of adult males and females [72]. Although vitellogenin functions in other species include hormone signaling, innate immune responses and pathogen recognition receptors, the function in the intestine of parasitic nematodes remains poorly understood [72].

We also considered the *A. suum* intestinal cell subpopulations identified at higher resolution clustering (7-CM), in order to determine whether there may be additional relevant biological complexity within the major clusters. Subcluster 1b is primarily defined by 283 genes with low expression compared to 1a (Supplementary Table S3f), with only 7 genes higher in 1b (Supplementary Table S3g). The functions associated with these 283 genes are enriched in multiple metabolic processes, along with encoding proteins such as Vitellogenin and Lipid transport proteins. Subcluster 1c is defined by low expression of genes associated with certain nitrogen metabolic processes and high expression of some C-type lectin genes (Supplementary Table S3h), while subcluster 1d shows high expression of genes involved in metabolism of chitin and other amino sugars (Fig. 4b; Supplementary Table S3i). Similarly, the relatively large subcluster 2b is defined by high expression of lipid transport, Vitellogenin and C-type lectin genes, compared to subcluster 2a (Fig. 4b; Supplementary Table S3j). Although the authenticity of subclusters in the 7-CM as distinct cell types is less clear than with clusters resolved in the 3-CM, these results should aid in further investigation of this issue.

We also considered the *A. suum* intestinal cell subpopulations identified at higher resolution clustering (7-CM), in order to determine whether there may be additional relevant biological complexity within the major clusters. Subcluster 1b is primarily defined by 283 genes with low expression compared to 1a (Supplementary Table S3f), with only 7 genes higher in 1b (Supplementary Table S3g). The functions associated with these 283 genes are enriched in multiple metabolic processes, along with encoding proteins such as Vitellogenin and Lipid transport proteins. Subcluster 1c is defined by low expression of genes associated with certain nitrogen metabolic processes and high expression of some C-type lectin genes (Supplementary Table S3h), while subcluster 1d shows high expression of genes involved in metabolism of chitin and other amino sugars (Fig. 4b; Supplementary Table S3i). Similarly, the relatively large subcluster 2b is defined by high expression of lipid transport, Vitellogenin and C-type lectin genes, compared to subcluster 2a (Fig. 4b; Supplementary Table S3j). Although the authenticity of subclusters in the 7-CM as distinct cell types is less clear than with clusters resolved in the 3-CM, these results should aid in further investigation of this issue.

To summarize major points of this 3-CM analysis, we identified (i) a cluster of *A. suum* intestinal cells (cluster 3) that displays most congruence with *C. elegans* intestinal cells, (ii) a cluster (cluster 1) that shows less, but still substantial, congruence with *C. elegans* intestinal cells and relatively close association with cluster 3, and (iii) a cluster (cluster 2) that displays little congruence with *C. elegans* intestinal cells and *A. suum* intestinal cells in clusters 1 and 3. The percentage of total cells that comprise each of these *A. suum* clusters indicates that each is a major constituent of the intestinal segment analyzed. While we expect that *A. suum* cells of clusters 1 and 3 have intestine-centric functions in common with intestinal cells of nematodes from across the phylum, the biological role(s) that cluster 2 cells fulfill is less clear. Based on these findings and available information on nematode intestinal cells, the transcriptional phenotype of cluster 2 cells is unique. More specifically, transcripts encoding many intestinal lumen-, apical intestinal membrane- and other prominent membrane-proteins documented for *A. suum* intestinal tissue [63] are not enriched among DE genes in cluster 2, but are in both clusters 1 and 3 (Fig. 3c). This finding is unexpected given that many of the under-represented transcripts encode hydrolases that are expected to function in the intestinal lumen as digestive enzymes, suggesting that cells in cluster 2 function in an unknown capacity compared to that perceived as a more classic nematode intestinal cell. As compared to the 20 cells that comprise the adult *C. elegans* intestine, the millions of intestinal cells in adult *A. suum* may have allowed compartmentalization of functions among intestinal cells to evolve towards greater exclusivity of key functions. Concurrently, specialization of *C. elegans* intestinal cells may have gone undetected when relying on classic intestinal cell markers to identify intestinal cells in the *C. elegans* study. Further it may be important that L2 were the subject of the *C. elegans* study and additional phenotypic diversity may exist among adult *C. elegans* intestinal cells. Another possibility is that cells in *A. suum* cluster 2 are undergoing a dynamic process that temporarily suppresses expression of more classic phenotypic markers. For instance, expression of differentiated cell products can be reduced or prevented while cells enter the cell cycle, and while it seems unlikely that such a large population of intestinal cells in adult *A. suum* would be undergoing cell cycle progression, we assessed this possibility in the next section.

### Cell cycle assessment and *A. suum* intestinal cell clusters

For the *A. suum* intestinal tract to grow and possess millions of cells in a mature adult, cell proliferation is a prerequisite. Hence, intestinal cell proliferation is expected during growth to the adult stage but has not been thoroughly investigated and demonstrated in *A. suum*. The extent to which intestinal cell proliferation occurs in mature adults for the purpose of cell replacement or repair also remains an open question. Nevertheless, previous assessment of millions of stained nuclei in context of normal and toxin-damaged adult intestine [7] and in the segment of intestinal tissue investigated here (Supplementary Fig. S1; *see* Methods) failed to reveal mitotic figures. Consequently, morphologic evidence is lacking for a large population of intestinal cells undergoing replication in the segment of *A. suum* intestine analyzed here.

To avoid potential confounding of tissue heterogeneity due to transcriptional signatures associated with cell replication cycle, potential proliferating cells were identified using Seurat’s ‘cellcyclescoring’ function [41], which compares the expression of a given set of S-phase associated genes (39 genes; Supplementary Table S3), and G2/M-phase associated genes (45 genes; Supplementary Table S4) [47], matched by BLAST searches. S-phase or G2/M-phase scores were assigned per cell, with unscored cells being considered G1 phase [41]. To adjust the data for differences in cell cycle phase, the normalized data of each sample was rescaled by regressing out the cell-cycle signal using Seurat.

While potential cell-cycle transcriptional genes were removed before clustering and analyzing DE genes in the analysis described above, S phase and G2/M marker genes (S4) were used to identify cells potentially in S, G2 or M phases among the three major *A. suum* clusters. The default cell cycle phase annotation is based on strict thresholds, with possible ambiguous, low-confidence annotations being assigned near the default thresholds. This is illustrated in Supplementary Fig. S6a. This initial approach detected > 43% of the cells annotated to express either S phase or G2/M phase genes, which could reflect active engagement in the cell cycle, with a higher number of cells annotated as such in cluster 2 (60.4%) compared to clusters 1 and 3 (31.6% and 33.5%, respectively; Supplementary Fig. S6b). To make this phase annotation more stringent, we incrementally increased the threshold to predict phase (i.e. a higher difference between S and G2/M scores). In this case, G2/M phase annotation shows a sharp decline for cluster 1 and 3 (Supplementary Fig. S6c), but not cluster 2 primarily due to a small number of cells from clusters 1 and 3 with high G2/M scores (see violin plot showing G2/M score distribution; Supplementary Fig. S6d). This sharp decline occurs near threshold 0.027. Thus, we chose to use this threshold for comparisons between cluster 2 and clusters 1 and 3.

Using this more stringent threshold, the percentage of all cells annotated to be in S and G2/M phases is ∼ 5.2% and ∼ 4.2%, respectively. At this threshold, cells with S and G2/M annotations are significantly more prevalent in cluster 2 (Supplementary Fig. S6e) (∼ 9% and ∼ 7.3%, *P*-values < 10^− 16^). However, mitotic figures have not yet been observed in many tens of thousands of nuclear stained cells from *A. suum* intestinal tissue (such as the image shown in Supplementary Fig. S1), so it is not clear that the modestly higher detection of predicted S and G2/M genes in cluster 2 cells accounts for the decidedly distinct transcriptional phenotype expressed by these cells compared to clusters 1 and 3.

### Cluster-specific heterogeneity in drug susceptibility and transcriptional responses to drug treatments

Motivated by the identified heterogeneity in *A. suum* untreated adult intestine based on our single-cell transcriptional analysis, and known bulk transcriptional differences associated with drug treatment response in *A. suum* [6, 7] and other nematodes [8], we aimed at comparing transcriptional response of two NITs at a single cell resolution. To that end, we repeated the single nuclear extraction procedure described above for 3 treatments– the NITs Leflunomide (LEF) and CID 106770

**Fig. 5:**
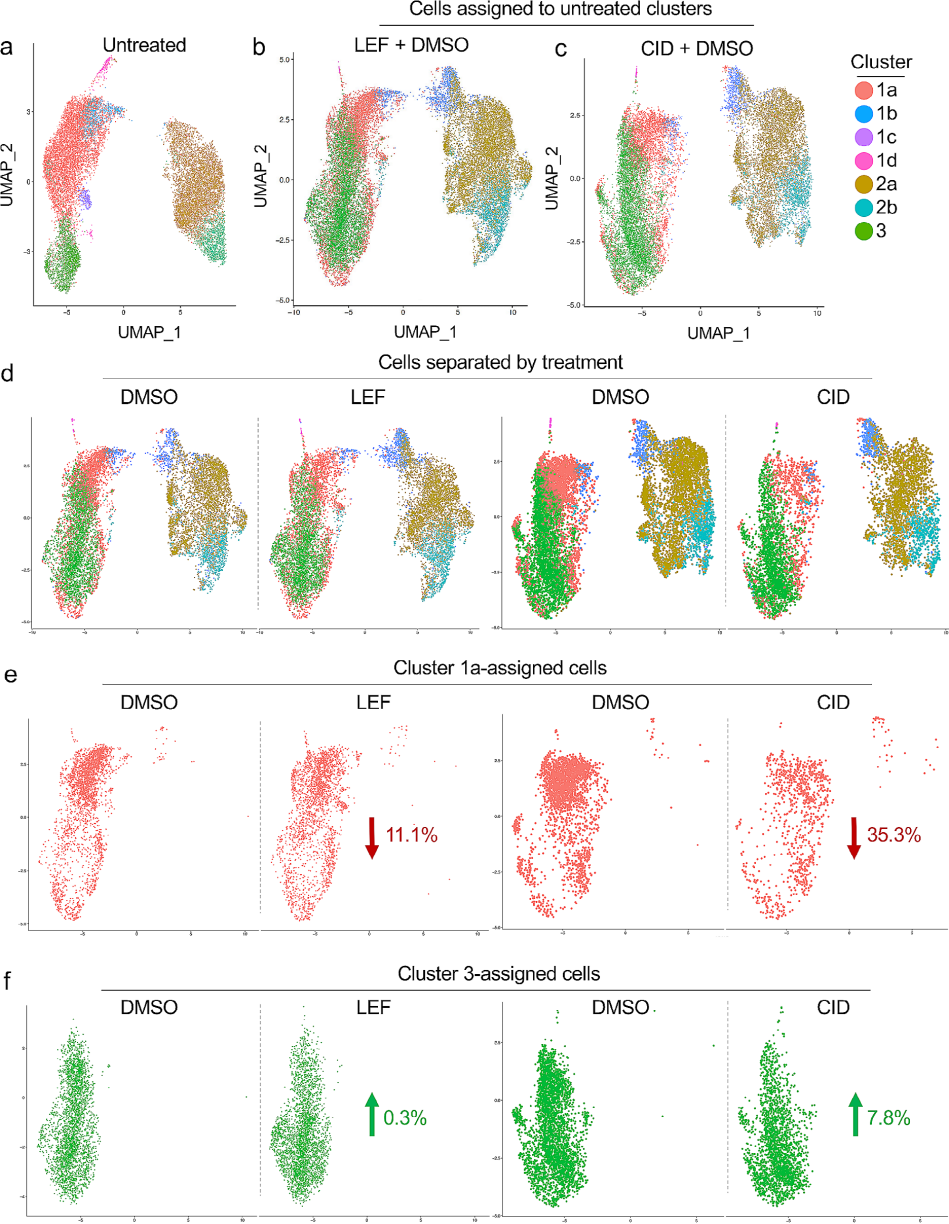
Changes in subcluster 1a and 3 relative cell abundance as a result of NIT treatment. a. Cluster numbering is based on the original 7-cluster analysis. UMAP plots of the LEF + DMSO samples (**b**) and the CID + DMSO samples (**c**) with cells colored according to cluster assignment to the untreated samples. **d**. In the treatment cohorts, cells are separated by DMSO vs. NIT treatment. **e**. The relative abundance of cells assigned to cluster 1a is reduced by 11.1% in the LEF clustering, and by 35.3% in the CID clustering, compared to DMSO. **F**. The relative abundance of cells assigned to cluster 3 is increased by 0.3% in LEF and 7.8% in CID, compared to DMSO

0 (CID), and the vehicle DMSO (each treatment repeated 2 times). For these samples, we were able to identify a median of 4022 cells each, having median 4423 UMIs per cell, resulting in median detection of 13,572 genes per sample (75.5% of *A. suum* protein coding genes; Table 1; Supplementary Table S1). To control for NIT treatment-independent changes, data from both NITs were integrated with DMSO-only data. To identify cells most closely associated with the major subpopulations of the untreated samples, we used the transcriptional profiles from the untreated tissue clustering analysis (Fig. 5a) as a reference to annotate the cells from the LEF and DMSO treatment (Fig. 5b) and the CID and DMSO treatment (Fig. 5c). This analysis showed that following DMSO, LEF and CID treatments, the overall intestinal transcriptional profiles remain largely unchanged, i.e. two major non-overlapping populations (superclusters as shown in Fig. 2) are identified (Fig. 5d). However, compared to DMSO, the NIT treatments reduced the relative proportion of subcluster 1a cells (by 11.1% with LEF and 35.3% with CID; Fig. 5e), while cluster 3 increased with both, but only substantially with CID (7.8%; Fig. 5f). The relative abundance of subcluster 2a cells was relatively unchanged with both treatments, although interestingly, small subcluster 1b did shift from a closer association with 1a to being associated with 2a in response to treatment.

**Fig. 6:**
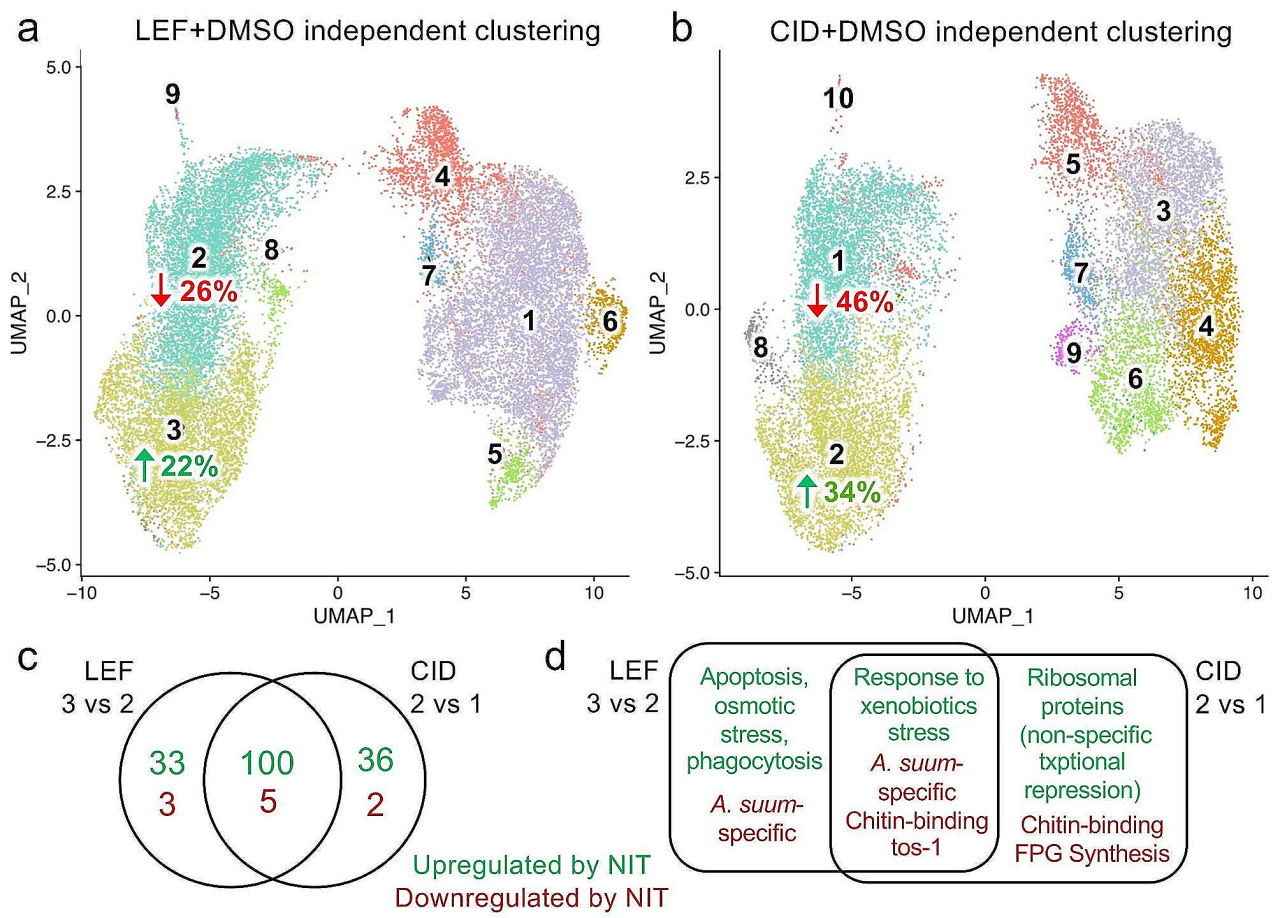
Comparison of cluster membership and differentially expressed genes from NIT treatments and DMSO-treated samples a. 9 clusters were identified in integrated data from Leflunomide treated sample and DMSO control, and the relative cell abundance of clusters 2 and 3 were reduced and increased (respectively) by LEF treatment. **b**. 10 clusters were identified in integrated data from CID 1067700 treated sample and DMSO control and the relative cell abundance of clusters 1 and 2 were reduced and increased (respectively) by LEF treatment. UMAP results are oriented to match the untreated sample layouts **c**. Intersection of genes differentially expressed among clusters differentially abundant following NIT treatments. **d**. Functional annotation of differentially expressed gene sets from panel **c**

To further bioinformatically explore this finding, each of the integrated datasets (LEF + DMSO and CID + DMSO) were independently clustered using the same resolution that was used for untreated tissue sample subcluster analysis (0.4), yielding 9 clusters for LEF (Fig. 6a) and 10 clusters for CID (Fig. 6b). As with the untreated reference-guided clustering (Fig. 5), independent clustering of DMSO and each NIT also demonstrated large NIT-induced shifts in the relative proportions of the corresponding clusters of the first major supercluster; LEF treatment reduced cluster 2 cells by 26% but increased cluster 3 by 22%, (Fig. 6a) and CID treatment reduced cluster 1 cells by 46% and increased cluster 2 by 22% (Fig. 6b). Gene expression profiles in these cluster pairs were used to identify significant [48] NIT-induced changes in gene expression (Fig. 6c). In both cases, we identify many more genes overexpressed with NIT treatment than those with suppressed expression compared to DMSO (133 genes higher with LEF vs. DMSO, Supplementary Table S3k; 8 genes lower with LEF, Supplementary Table S3l; 136 higher with CID, Supplementary Table S3m; 7 genes lower with CID, Supplementary Table S3n). It is worth noting that in both cases, the treatment associated-cluster (cluster 3 for LEF and cluster 2 for CID) is significantly similar to untreated cluster 3 (Fisher test *P*-values < 2 × 10^− 16^) and the DMSO associated cluster (cluster 2 for LEF and cluster 1 for CID) to untreated cluster 1 (Fisher test *P*-values < 2 × 10^− 16^) based on their transcriptional profile.

Remarkably, 100 of these overexpressed genes are common to both of the NIT treatment responses (Fig. 6c), suggesting a set of common stress-responses. There are multiple functions annotated to these 100 genes (Fig. 6d, Supplementary Table S3o), including carbohydrate metabolism and absorption and lipid transport, which have been previously implicated in metabolic state transition in response to xenobiotic stress [73]. Specifically, we identified glycosyl hydrolases and galactose mutarotases among this gene set. Multiple genes associated with lipid transport, including multiple vitellogenins, are also overexpressed, potentially due to ER stress induced by toxins, or cellular responses to the ER stress.

The 33 genes induced only in response to LEF treatment (Supplementary Table S3p) include multiple stress or apoptosis associated genes including cadherin [74, 75], clathrin [76, 77], calreticulin [78–80] and multiple ubiquitin-pathway genes [81–84]. One of these 33 genes, AgR005_g305, annotated as “olpB: C-type lectin protein 160”, was also overexpressed in *A. suum* L3 larvae after four hours of treatment of LEF treatment (but not CID; [7]). On the other hand, there are multiple ribosomal proteins among the 36 genes induced only in response to CID treatment (Supplementary Table S3q; Supplementary Fig. S7), something not seen with LEF-treatment. This indicates possible differences in the toxicity mechanism and/or the cellular responses to these NITs, with LEF treatment involving specific pathways related to osmotic stress, phagocytosis and apoptosis. Another possibility is that at least some of the identified genes may be implicated in making those cells more susceptible to the toxin compared to other cell populations, rather than being directly involved in the toxin stress mechanism or the cell’s stress response.

Additionally, among the small number of genes whose expression is suppressed upon these NIT-treatments also include 5 that may comprise general stress-response (Fig. 6c and d; Supplementary Table S3r). These include 2 *Ascaris*-specific genes, but also 2 chitin-binding genes and tos-1. “Target of splicing” (tos-1) is a direct target of the splicing factor SFA-1 [85], and has been used as a reporter of splicing in vivo [86] albeit its cellular function has not yet been determined and it has not directly been associated with a general stress response.

Further understanding of the molecular determinants of the heterogeneity of intestinal cell responses to NITs is likely to provide insights into their mechanisms of action, paving the way for better leveraging this novel set of potential anthelmintics.

### Concluding remarks

This investigation of single intestinal cell transcriptional responses to NITs illustrates advances in knowledge related to technical capabilities and biological differences among major cell populations of adult *A. suum* intestinal cells. The stepwise design and analysis on untreated intestinal cells, for the first time, resolved diverse cell populations and produced unique biological identifiers of each that were essential to support tracking of constituent cells under experimental conditions. The large number of intestinal cells detached from surrounding tissues obtainable from single *A. suum* adult worms has been another critical factor of this experimental system that increases statistical power to detect responses such as those demonstrated here.

The short duration of NIT treatments was intended to avoid complications of secondary and later responses, while also risking too little time to achieve detectable transcriptional responses. The rapidity with which intestinal cells responded to the direct experimental treatments diminished these concerns. However, there remains concern over a cascade of downstream response that may occur with prolonged treatment duration and complicate analysis of certain kinds of experiments. Nevertheless, both the computational and biological methods now available provide a platform to more deeply probe the biology of nematode intestinal cells with respect to anthelmintic treatments.

At the same time, results obtained here identify differences in transcriptional responses to toxic treatments among major cell populations differentiated within the *A. suum* intestine. Although it is currently unclear if these responses reflect relative sensitivity to pathologic anthelmintic activity, the findings promote ideas on how to test this hypothesis. The differences in responses among cells linked by derived markers to major superclusters I or II and associated clusters and subclusters of untreated cells also establish another biological feature that distinguishes supercluster II cells and its subclusters (less responsive to NIT treatments) from that of supercluster I cells and its subclusters with the exception of the small subcluster 1b (more responsive to NIT treatments), at least during the time frame of this experiment. While the possibility that supercluster II cells would have demonstrable responses with longer exposure cannot be excluded, the findings add support that supercluster II cells represent a biologically distinct cell population that may be an invention of nematodes belonging to Clade III of the phylum Nematoda.

The results also indicate that intestinal cells that belong to supercluster I and its subclusters (1a and 3) were most transcriptionally responsive to NIT treatments. It may be significant that even with this responsiveness, resulting phenotypes appeared to shift to subclusters contained within supercluster I, rather than form new clusters distinct from this supercluster. This behavior may indicate plasticity that is restrained by dominant determinants of supercluster I and its allied subclusters. Further verification of this possibility will be important toward identifying molecular determinants that may be involved.

It is also possible that one or more of the cluster phenotypes are not terminally differentiated. For instance, the phenotype of cluster 3 (most similar to *C. elegans* larval intestinal cells) may represent terminally differentiated cells, and cluster 1 may represent cells maturing toward terminal differentiation. Although distinctly different from clusters 1 and 3, cluster 2 could represent undifferentiated precursors to cluster 1 and/or 3 cells. Responses of clusters to NITs could be instructive here between clusters 1 and 3, with the inverse proportional increase and decreases observed between these two clusters potentially reflecting phenotypic switching after NIT treatment. On the contrary, the proportion of cells in cluster 2 did not noticeably change with NIT treatments, potentially indicating a developmentally distinct lineage of cells. Although insufficient to provide confidence in these possible explanations, available data do provide direction for further investigation.

### Electronic supplementary material

Below is the link to the electronic supplementary material.

Supplementary Material 1

Supplementary Material 2

## Data Availability

The sequencing datasets generated and analyzed during the current study are available in the NCBI Sequence Read Archive (SRA, BioProject PRJNA167264; https://www.ncbi.nlm.nih.gov/bioproject/167264), with accession numbers associated with each sample provided in Supplementary Table S1. Complete sample metadata, read counts, normalized expression values and differential expression statistics are provided in Supplementary Table S2. No custom code or novel mathematical algorithms were used in the generation of the dataset or the bioinformatic analyses. The specific programs and algorithms used are described in sufficient detail in the methods in order for the analysis to be replicated. All R scripts used to generate results have been made publicly available for download on GitHub: https://github.com/brucearosa/Asuum_Intestine_scRNAseq.

## Abbreviations

NIT: Nematode intestinal toxin
LEF: Leflunomide
CID: CID 1067700
3-CM: 3-cluster model
7-CM: 7-cluster model
DE: Differentially expressed
