# Supplementary material for: Intestinal cell diversity and treatment responses in a parasitic nematode at single cell resolution
Source: BMC Genomics. 2024 Apr 4;25:341. doi: 10.1186/s12864-024-10203-7 (PMC10996262; doi:10.1186/s12864-024-10203-7)
Supplement: Supplementary file 2 — Supplementary Material 2 [file 12864_2024_10203_MOESM2_ESM.pdf]

Intestinal cell diversity and treatment responses in a parasitic nematode at single cell resolution

Rahul Tyagi[1,5], Bruce A Rosa[1,5], Douglas P Jasmer[2,6], Makedonka Mitreva[1,3,4,6]

[1] Division of Infectious Diseases, Department of Internal Medicine, Washington University School of Medicine, St. Louis, MO 63110, USA

[2] Department of Veterinary Microbiology and Pathology, Washington State University, Pullman, WA 99164, USA

[3] Department of Genetics, Washington University School of Medicine, St. Louis, MO 63110, USA

[4] McDonnell Genome Institute, Washington University in St Louis, St Louis, MO, USA.

[5] These authors contributed equally.

[6] Corresponding authors.

Correspondence: mmitreva@wustl.edu; Tel.: +1-(314)286-2005

Emails:

Rahul Tyagi rahul.tyagi@gmail.com

Bruce A Rosa barosa@wustl.edu

Douglas P Jasmer djasmer@wsu.edu

Makedonka Mitreva mmitreva@wustl.edu

Supplementary Figures

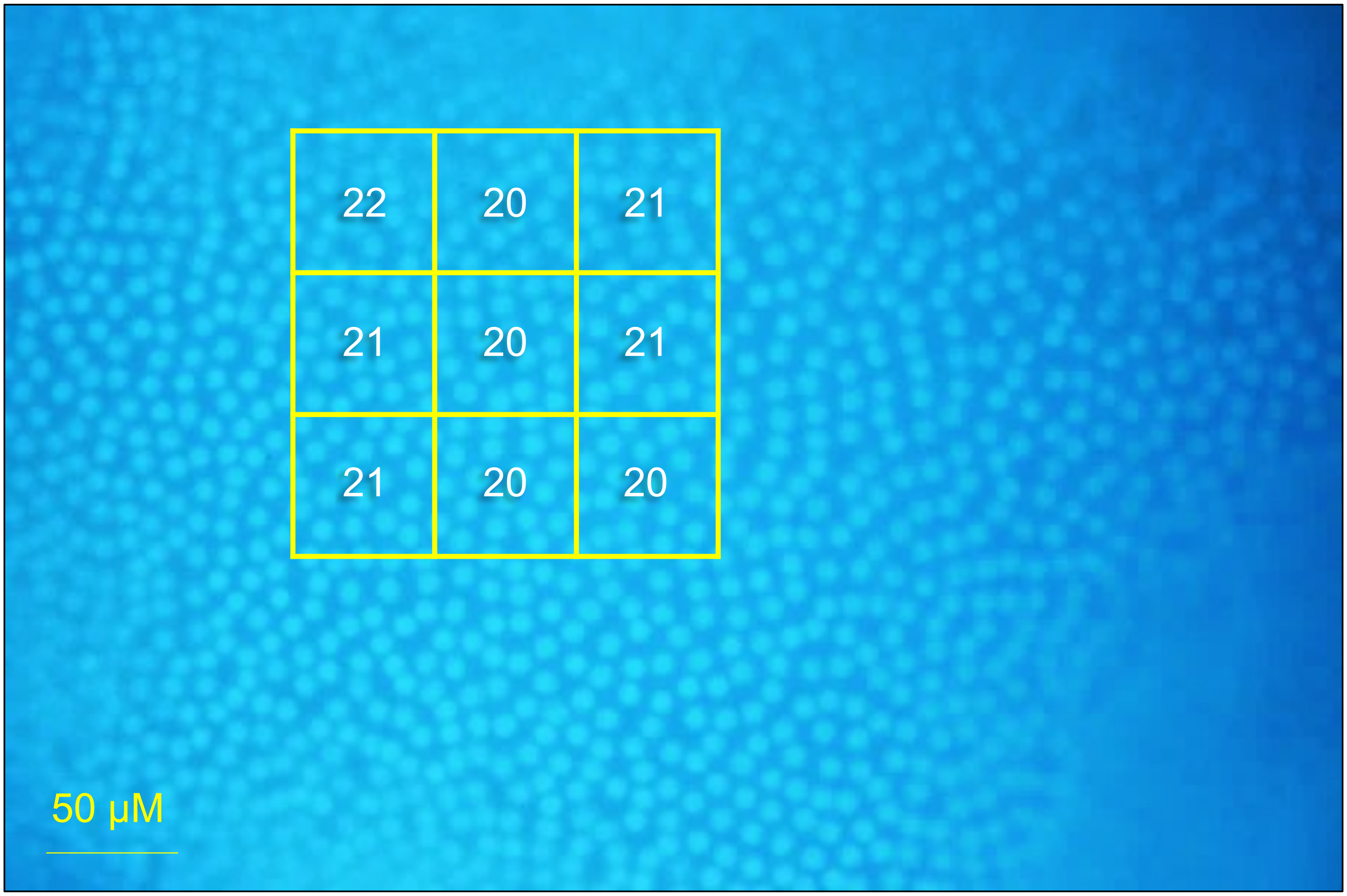

Supplementary Fig. S1 Intestine cell count estimate. Bisbenzimide-stained adult female *A. suum* intestine image used to estimate total cell count per intestine. An average of 20.667 cells were counted per 50µM x 50µM area, 826,680 cells per cm^2 and 8,266,800 cells per intestine, based on 10cm length, 0.5cm width and 2 sides to the intestine.

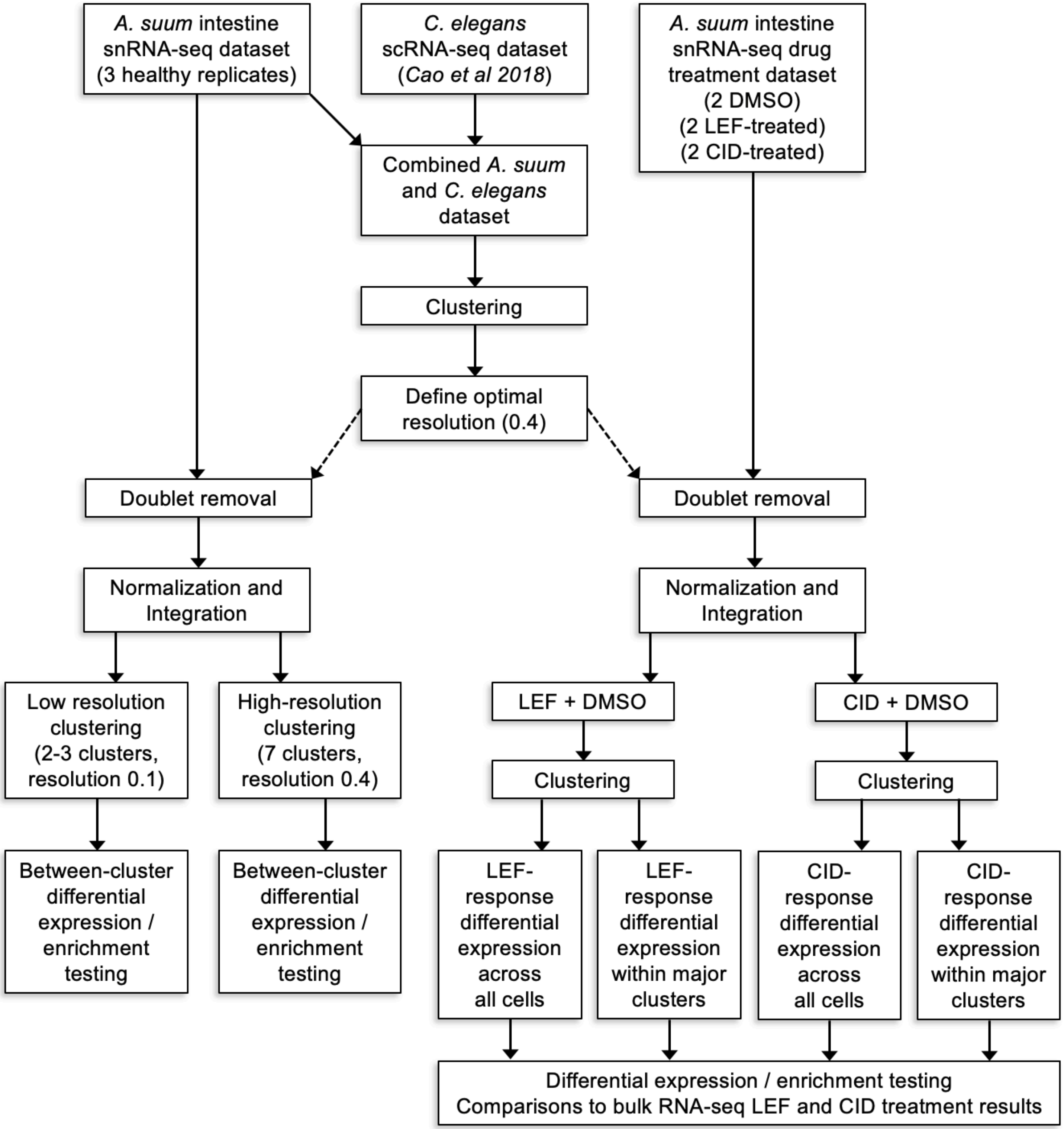

Supplementary Fig 2 Flowchart describing the overall workflow for the scRNA-seq analysis.

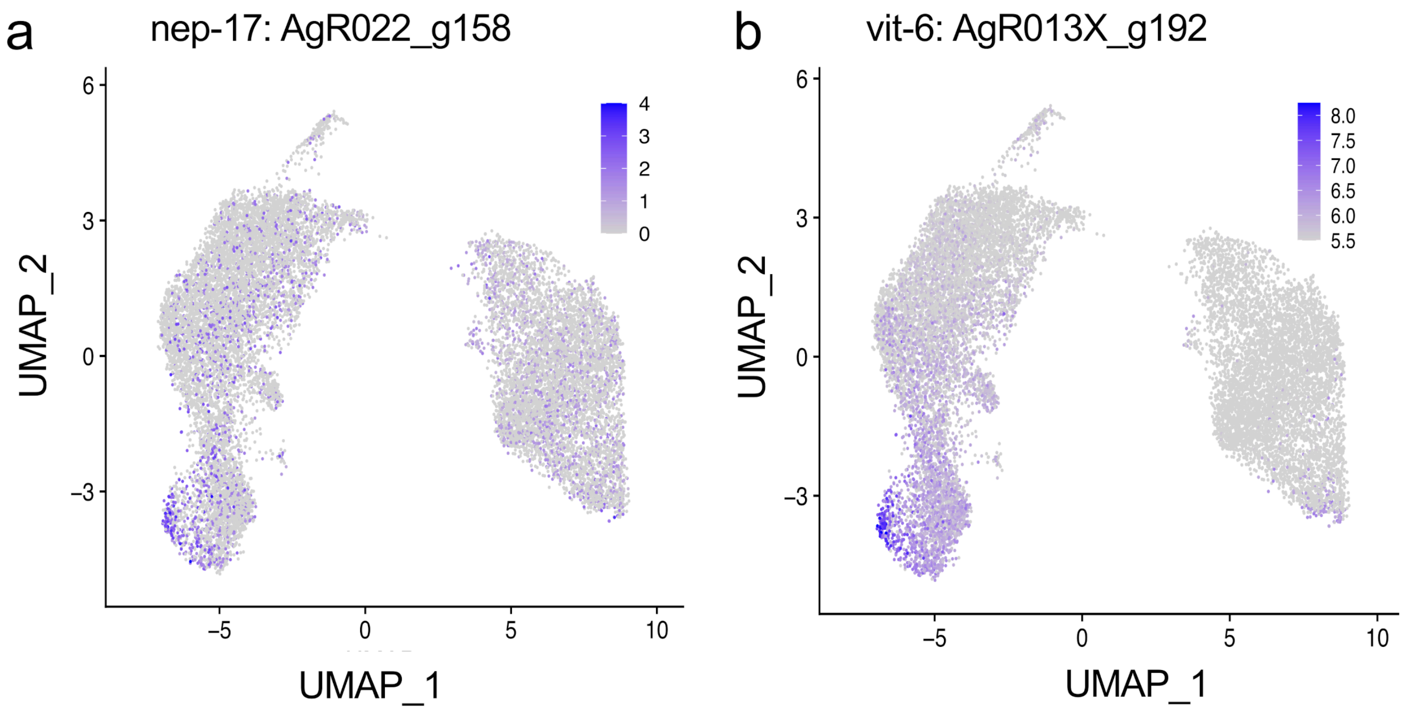

Supplementary Fig. S3 Genes previously used as *C. elegans* intestinal markers including (a) nep-17 and (b) vit-6 show cluster 3-enriched expression. Note that vit-6 expression in panel b has a minimum expression cutoff applied to the scale, to better visualize cluster-3 enrichment.

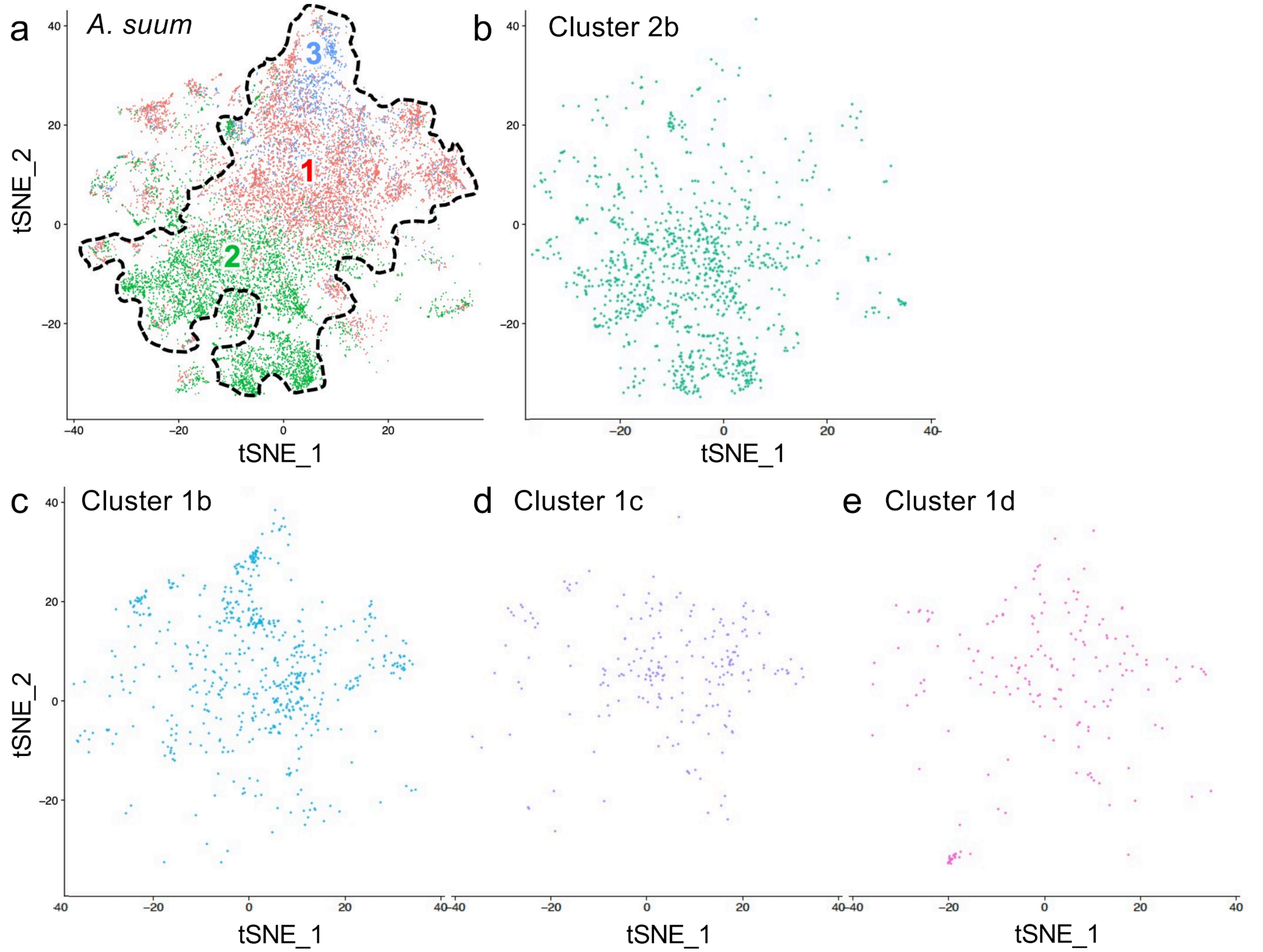

Supplementary Fig. S4 ***A. suum*** **untreated tissue-derived cells plotted on coordinates based on** ***C. elegans*** **integrated data (Fig. 1) and separated by** ***A. suum*****-only subpopulation clusters (Fig. 2). a**. Colors depicting cells from the 3 major clusters. Cluster 3 subpopulation is collocated with intestinal cells from *C. elegans* (**Fig. 1e**). **b-e**. Smaller subcluster cells separated out for clarity, for clusters 2b (**b**), 1b (**c**), 1c (**d**) and 1d (**e**).

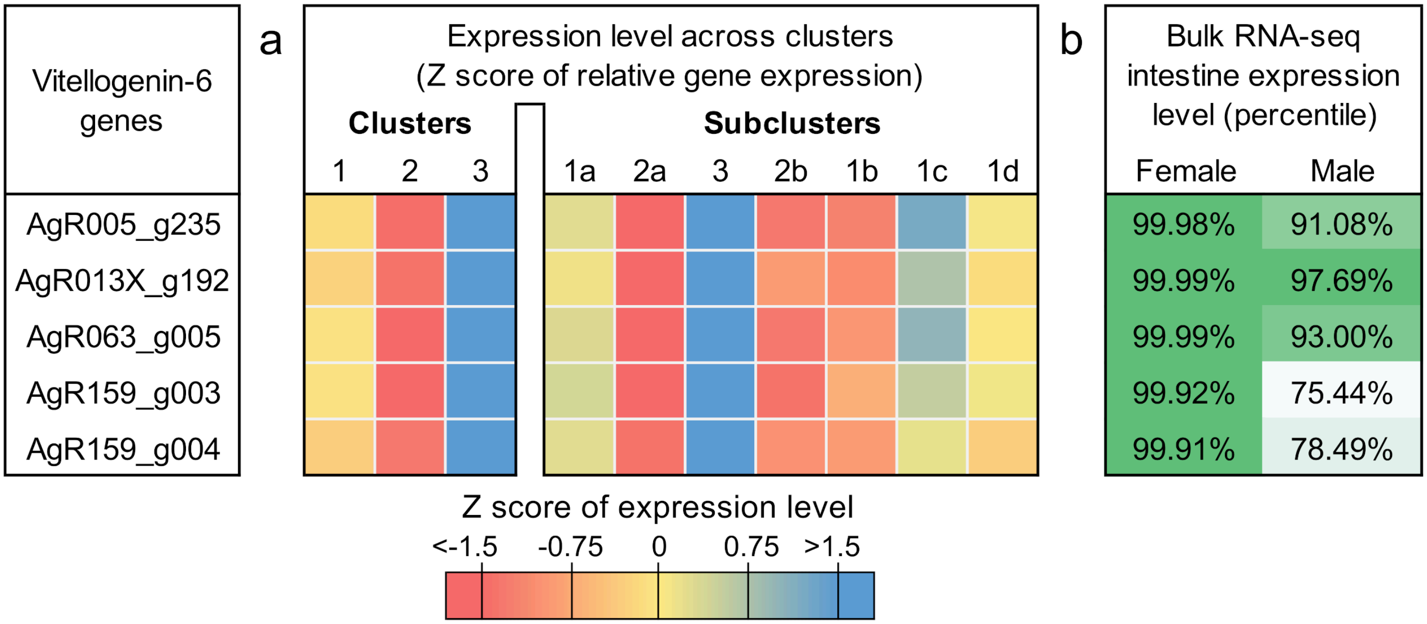

Supplementary Fig. S5 Relative gene expression levels of *A. suum* vitellogenin-6 genes. **a**. Z-score normalized expression levels show highly correlated expression across clusters and subclusters in the scRNA-seq analysis. **b**. The percentile of the expression level of the vit-6 genes in adult female and male intestine bulk RNA-seq samples (based on Rosa et al, 2014). For example, AgR005_g235 was more highly expressed than 99.98% of all expressed genes in the adult female intestine.

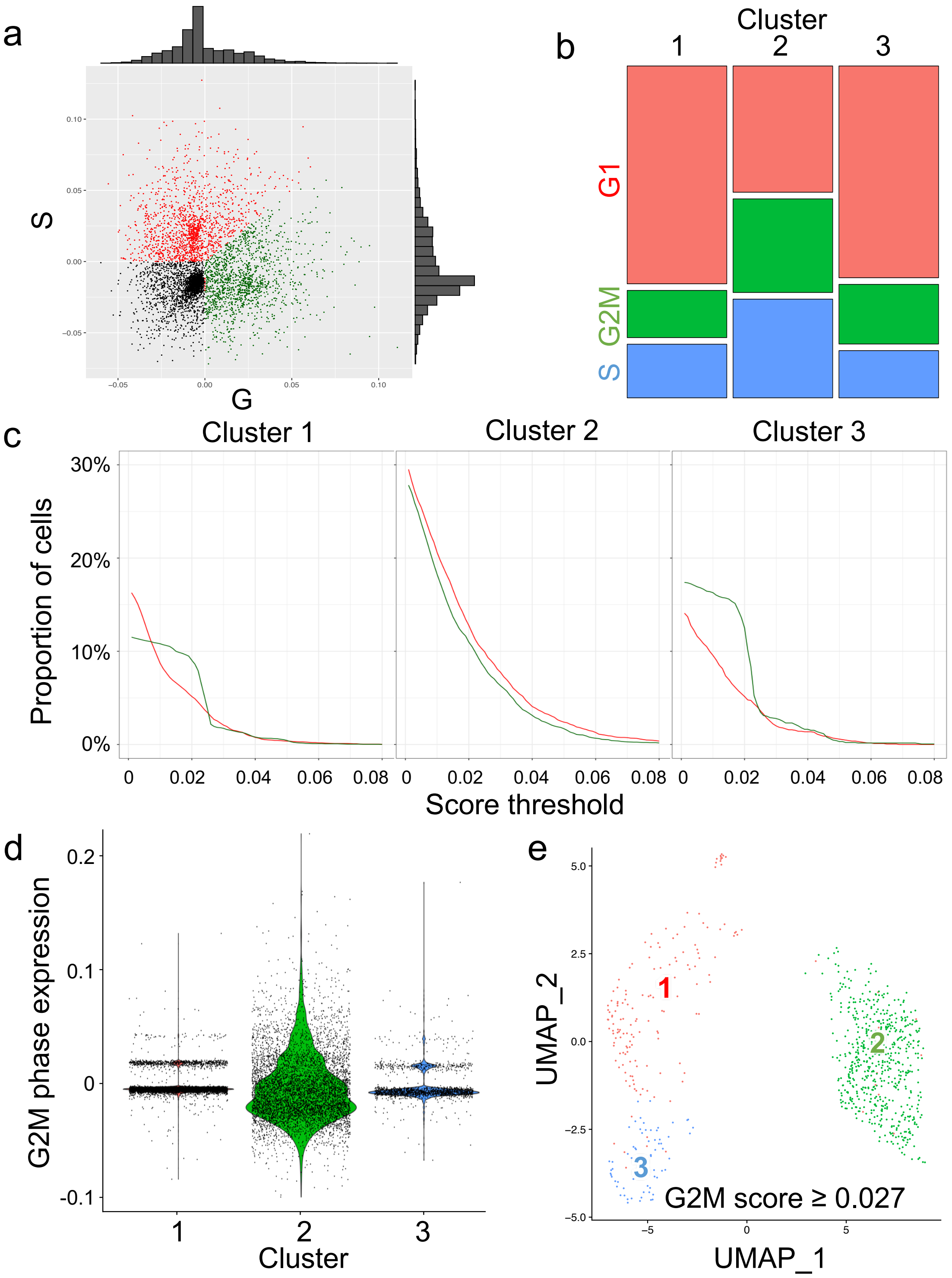

Supplementary Fig. S6 Cell cycle phase annotation. (**a**) Annotation based on strict thresholding on S-phase and G2M-phase scores. Histograms on X and Y axes show cell counts. Red = S-phase cells, green = G2M-phase cells. **b**. Count of cells annotated as belonging to different phases, based on panel A. **c**. Impact on Cell cycle phase annotation depends on the difference between S and G2M scores required for confident annotation. Cluster 2 shows a much larger population of cells with high G2M score. Red = S-phase, green = G2M-phase. **d.** distribution of G2M score shows cluster 2 cells have a different expression profile of G2M associated genes. **e**. UMAP showing that cells with high G2M score (≥ 0.027) are preferentially associated with cluster 2.

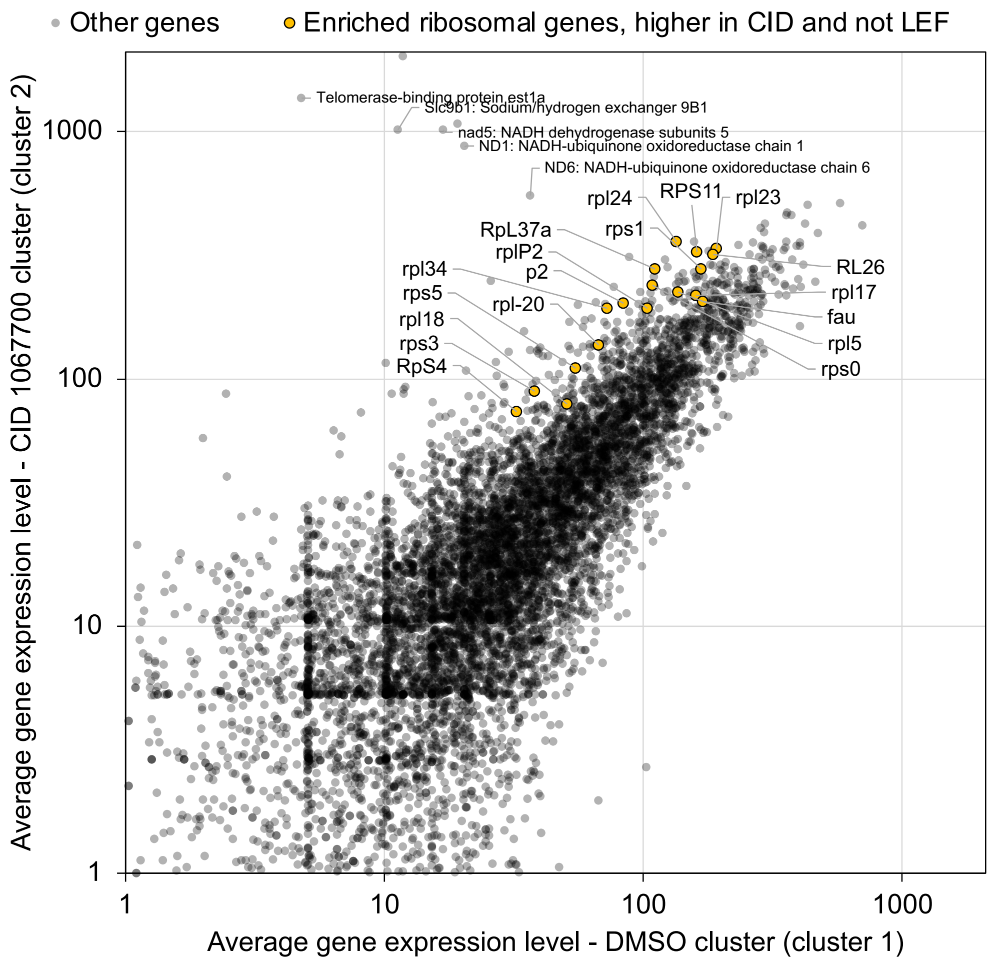

Supplementary Fig. S7. The 18 ribosomal genes (orange, with labels) among the 36 genes significantly higher in CID 106770 vs DMSO (cluster 2 vs cluster 1) and not in Leflunomide vs DMSO (cluster 3 v2) vs DMSO. Genes are plotted according to their log-scale average expression level in the clusters with higher abundance in DMSO (cluster 1) and CID 106770 (cluster 2). Some additional genes with substantial differential expression are labeled at the top of the plot.

Supplementary Tables (saved in separate MS Excel file)

Supplementary Table S1: NCBI SRA accession IDs, Cell Ranger cell, gene and read count statistics, and processed and filtered sample statistics for each of the scRNAseq samples. UMI = unique molecular identifier.

Treatment cohort	Untreated			DMSO-treated		CID 1067700-treated		Leflunomide-treated	
Sample	F1_UT	F2_UT	F3_UT	DMSO_1	DMSO_2	CID_1	CID_2	LEF_1	LEF_2
SRA accession (BioProject PRJNA167264)	SRR160 21934	SRR160 21933	SRR160 21932	SRR160 21938	SRR160 21937	SRR160 21940	SRR160 21939	SRR160 21936	SRR160 21935
**Cell Ranger output**									
Estimated number of cells	7,757	10,563	8,104	4,706	6,219	3,684	2,641	3,739	7,291
Number of reads (million)	665.4	492.0	552.7	497.8	480.4	449.8	448.3	509.4	565.0
Mean reads per cell	85,784	46,575	68,197	105,782	77,252	122,106	169,737	136,232	77,490
Median genes per cell	687	285	203	327	1,054	480	1,279	243	882
Fraction of reads in cells	43.2%	39.3%	37.3%	29.0%	32.6%	34.0%	40.5%	39.3%	41.7%
Total genes detected	14,024	11,800	12,192	13,832	12,976	13,024	13,501	13,686	13,643
Median UMI counts per cell	3,883	2,249	1,415	3,180	4,585	4,262	8,635	1,906	5,910
**Filtered statistics**									
Number of cells	4,980	7,102	3,047	4,706	6,218	3,684	2,641	3,661	7,291
Mean UMIs per cell	4,670	3,232	3,048	5,589	5,571	6,979	9,842	5,947	7,244
Median genes per cell	852	224	409	813	1,128	1,068	1,325	811	1,093

Supplementary Table S2: Complete database of *A. suum* gene functional annotations, differential expression statistics for each cluster-based comparison, and re-analyzed datasets from previous studies.

Supplementary Table S3: Differentially expressed gene function annotation and enrichment for all cluster comparisons of interest. If no enrichment is indicated, then no significantly enriched KEGG, gene ontology or InterPro domains were identified for a gene set. Figures shown at the left identify the associated gene sets, for reference.

Supplementary Table S4: S-phase and G2/M-phase genes based on orthologs of human genes, used for cell cycle normalization (as described in the Methods).
